# Site‐Specific Encoding of Photoactivity in Antibodies Enables Light‐Mediated Antibody–Antigen Binding on Live Cells

**DOI:** 10.1002/anie.201908655

**Published:** 2019-10-31

**Authors:** Thomas Bridge, Saher A. Shaikh, Paul Thomas, Joaquin Botta, Peter J. McCormick, Amit Sachdeva

**Affiliations:** ^1^ School of Chemistry University of East Anglia Norwich NR4 7TJ UK; ^2^ The Henry Wellcome Laboratory of Cell Imaging University of East Anglia Norwich NR4 7TJ UK; ^3^ Centre of Endocrinology William Harvey Research Institute Queen Mary University London Charterhouse Square London EC1M 6BQ UK

**Keywords:** antibodies, cancer, protein design, synthetic biology, unnatural amino acids

## Abstract

Antibodies have found applications in several fields, including, medicine, diagnostics, and nanotechnology, yet methods to modulate antibody–antigen binding using an external agent remain limited. Here, we have developed photoactive antibody fragments by genetic site‐specific replacement of single tyrosine residues with photocaged tyrosine, in an antibody fragment, 7D12. A simple and robust assay is adopted to evaluate the light‐mediated binding of 7D12 mutants to its target, epidermal growth factor receptor (EGFR), on the surface of cancer cells. Presence of photocaged tyrosine reduces 7D12‐EGFR binding affinity by over 20‐fold in two out of three 7D12 mutants studied, and binding is restored upon exposure to 365 nm light. Molecular dynamics simulations explain the difference in effect of photocaging on 7D12‐EGFR interaction among the mutants. Finally, we demonstrate the application of photoactive antibodies in delivering fluorophores to EGFR‐positive live cancer cells in a light‐dependent manner.

## Graphical abstract

**Site‐specific installation** of a single photoactive amino acid in an antibody enables development of photoactive antibodies allowing light‐controlled, cell‐specific delivery of small molecules on live cancer cells.

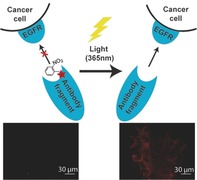

## Introduction

Chemists and biochemists have successfully designed molecular systems that can be controlled in a defined manner in response to external agents, such as pH, light, and small molecules.^1^ Controlling the activity of small molecules and biomolecules has allowed development of molecular machines, novel drugs, and nano‐delivery systems, that have found widespread applications.^2^ Monoclonal antibodies are arguably one of the most versatile biomolecules that can be adapted to bind to different substrates with high selectively and specificity. Due to these properties, antibodies have found applications as building blocks in molecular electronics, as agents for detection of substrates in medical diagnosis and biotechnology, and as inhibitors of biological processes in biotherapeutics.^3^

Modulating antibody–antigen binding presents an opportunity to gain user‐defined control over antibody‐mediated processes. Despite immense potential, there are only a few reports on controlling the binding of antibodies to their target. Notable examples are, antibodies activated by tumor‐specific proteases, and those activated by phosphatases. The former are currently under investigation for cancer therapy, and are generated by extending the N‐terminal domain of the antibody.^4^ The latter have been generated by chemically attaching phosphate to cysteine in an antibody fragment.^5^ These approaches are restricted by the availability of sites for inhibitory groups, and dependent on addition of the activating enzyme. A method where a controllable functional group can be incorporated at any site in an antibody, would allow wide applicability. In addition, adopting light as an activator, would present the opportunity to gain spatial and temporal control over antigen‐antibody binding in a facile manner, independent of other molecules.

Selective therapeutic targeting of cells is a major challenge in medicine, particularly in cancer therapy. Light‐activated small molecule cytotoxic drugs are currently under investigation for treatment of cancer, that could target cells in a localized area.^6^ However, after photoactivation these drugs are often not cell‐selective, and could cause side effects. Many antibodies and antibody–drug conjugates (ADCs) are in use, or in clinical trials, for treatment of cancer.^7^ These antibodies exert cytotoxicity by binding and blocking the function of receptors on the surface of cancer cells, and in the case of ADCs, also delivering cytotoxic drugs to cancer cells. As the same cell surface receptors are often present on healthy cells, therapeutic antibodies can have severe side effects.^8^ To partly address this challenge, antibodies linked to light‐activated small molecule drugs have also been developed.^9^ However, the antibody would still be able to bind to healthy cells independent of light. Light‐activatable antibodies have also been generated by non‐specific coating of antibodies with 1‐(2‐nitrophenyl)ethanol using a chemical method.^10^ However, this method generates non‐homogeneously labelled antibody samples, limiting future therapeutic applications. Site‐specific modification of antibodies would allow development of homogeneous therapeutic antibodies. Developing such homogenously modified antibodies, where antigen binding could be directly controlled using light, at the site of cancer, would be useful to minimize the side effects of antibody‐based therapeutics.

Over the last two decades, genetic code expansion has enabled site‐specific incorporation of unnatural amino acids, including amino acids containing bioorthogonal functional groups, photoreactive amino acids and photocaged amino acids, into proteins.^11^ Photocaged amino acids, in particular, have been employed to control the activity of several biomolecules including DNA polymerase,1d RNA polymerase,^12^ kinases,^13^ proteases^14^ and inteins,^15^ which have undoubtedly advanced our understanding of key biological processes. To the best of our knowledge, site‐specifically incorporated photocaged amino acids have not been used to control the activity of therapeutically significant antibodies. In the present study, we show that modifying a single amino acid to its photocaged counterpart in the antigen binding region of an antibody fragment, 7D12, inhibits its binding to its target, epidermal growth factor receptor (EGFR). EGFR is overexpressed in several cancers, including colorectal cancer, lung cancer, and head and neck cancer. Therapeutic antibodies that bind to the extracellular domain of EGFR, block its downstream signaling and inhibit cell growth;^16^ however, these can cause severe side effects.^17^ 7D12 belongs to a class of single domain antibody fragments isolated from camelids that have gained importance due to their small size and deep tissue penetration,^18^ and has shown promise in treatment of cancers in mice model.^19^

Here, we demonstrate efficient genetic site‐specific incorporation of photocaged tyrosine (**pcY**) into 7D12, generating photoactive antibodies. Using an on‐cell assay, we show that the presence of a photocaging group at specific tyrosine residues in the antigen binding region of 7D12 inhibits its binding to EGFR on the surface of cancer cells and the binding is restored upon irradiation with 365 nm light. In order to explain why the binding of 7D12 to EGFR is affected by **pcY** at only certain positions at the binding interface, we investigated the 7D12‐EGFR interaction using molecular dynamics simulations. Finally, we show that these photoactive antibodies mediate light‐dependent delivery of small molecule fluorophores to the surface of EGFR‐positive live cancer cells.

## Results and Discussion

**Efficient genetic site‐specific incorporation of photocaged tyrosine into antibody fragments**. Wild‐type 7D12 (wt7D12) was cloned into pSANG10 plasmid,^20^ (Page S3 and Figure S1) and expressed in *E. Coli*, resulting in a high yield (10.1 mg of wt7D12 per litre of culture, Figure S2); pSANG10 has earlier been employed for efficient expression of single chain antibody fragments in the periplasm of *E. coli*.^20^

To design photoactive mutants of 7D12, we aimed to replace tyrosine residues with **pcY** in the antigen binding site of 7D12. Mutants of *M. jannaschii* Tyrosyl‐tRNA synthetase (*Mj*RS)/*Mj*tRNA pair and *Methanosarcina* Pyrrolysyl‐tRNA synthetase (PylRS)/tRNA pair have been employed earlier to genetically encode **pcY**.^21^ Several suppressor plasmids are known, that contain orthogonal aminoacyl‐tRNA synthetase (aaRS)/tRNA pairs for incorporation of unnatural amino acids in response to an amber (TAG) stop codon, in *E. coli*.^22^ These plasmids vary in their origin of replication, promotors that drive the expression of aaRS and tRNA, and the copy number of aaRS and tRNA genes. To find an optimal plasmid system and aaRS/tRNA pair for incorporation of **pcY** in 7D12, we screened five suppressor plasmids containing either *Mj*CNFRS/*Mj*tRNA_CUA_ pair (*Mj*CNFRS is an *Mj*RS evolved for incorporation of 4‐cyano‐l‐phenylalanine) or the PylRS/tRNA_CUA_ pair (Page S3 and S4, and Figure S3 and S4). pULTRA plasmid with *Mj*CNFRS/*Mj*tRNA_CUA_ pair, and pCDF plasmid with PylRS/tRNA_CUA_ pair show most efficient genetic incorporation of unnatural amino acids. Due to the ease of cloning, we selected pULTRA and the *Mj*CNFRS/*Mj*tRNA_CUA_ pair, replacing the *Mj*CNFRS with *Mj*pcYRS (aaRS evolved for **pcY**) (Page S3).

**Figure 1:**
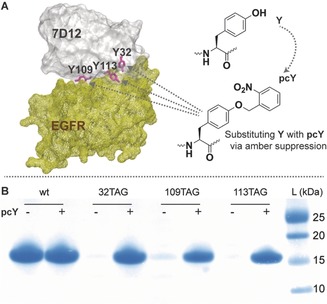
Genetic site‐specific incorporation of **pcY** in 7D12. A) Crystal structure of 7D12 (grey)–EGFR domain III (yellow) complex (PDB ID: 4KRL)^23^ showing Y32, Y109, and Y113 (pink) in the antigen binding pocket of 7D12, that were replaced with **pcY**. B) The expression of three amber mutants of 7D12, viz. 32TAG, 109TAG and 113TAG only occurs in the presence of **pcY**. Comparison of band intensities for amber mutants with wt7D12 shows efficient incorporation of **pcY**.

Examining the crystal structure of 7D12 bound to domain III of EGFR (PDB ID: 4KRL),^23^ we identified three tyrosine residues in the antigen binding site of 7D12, viz. Y32, Y109 and Y113, as candidates for developing photocaged mutants (Figure 1 A). These were replaced with **pcY** by assigning amber stop codon, TAG, to these positions, forming the mutants, 7D12**pcY**32, 7D12**pcY**109, and 7D12**pcY**113, respectively. Protein expression was performed both, in the presence, and absence, of **pcY**. For the amber mutants, expression of full‐length protein was observed only on addition of **pcY** (Figure 1 B). High yields of amber mutants with **pcY** were obtained after purification: 5.3 mg of 7D12**pcY**32, 3.2 mg of 7D12**pcY**109, and 1.7 mg of 7D12**pcY**113, per litre of culture. Electrospray ionization mass spectrometry (ESI‐MS) analysis of full‐length 7D12 and the mutants was consistent with incorporation of **pcY** (Figure S5).

**Figure 2:**
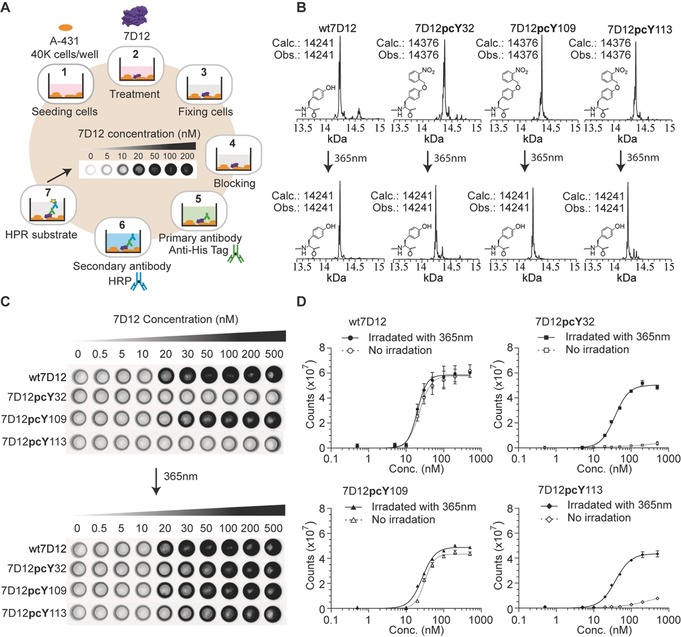
Assessing the binding of photocaged mutants of antibody fragment to EGFR on cell surface. A) Schematic representation of procedure followed for measurement of 7D12–EGFR binding on the surface of A431 cancer cells. 1. 40 000 cells were seeded into each well of a 96‐well plate. 2. These cells were incubated with the complete media containing the antibody fragment. 3. The antibody solution was replaced with 3.7 % formaldehyde solution for fixing the cells. 4. Incubation with blocking solution. 5. Incubation with primary antibody specific for hexa‐histidine tag. 6. incubation with HRP‐linked secondary antibody. 7. The substrate for HRP was added and the cells were imaged for chemiluminescence (Page S5). B) Comparison of ESI‐MS of photocaged mutants of 7D12 before and after irradiation with 365 nm light confirms light‐mediated decaging. See Page S6 for decaging conditions. C) The on‐cell binding assay demonstrates that the presence of **pcY** at positions 32 and 113 in 7D12 inhibits its binding to EGFR. 7D12**pcY**109 mutant does not show inhibition in binding to EGFR. Binding of 7D12**pcY**32 and 7D12**pcY**113 is restored upon irradiation with 365 nm light. These experiments were performed in triplicate (Figure S8). D) Chemiluminescence intensity was quantified using a CLARIOstar plate reader and plotted against concentration of 7D12, where X‐axis is in log scale; the data was fitted to a sigmoidal nonlinear curve using GraphPad (Figure S9). Some error bars are too small to be clearly visible.

**Assessing the binding of photoactive antibodies to EGFR on the surface of cancer cells**. To study 7D12‐EGFR binding, we adopted an assay that would report on this interaction in a cellular environment where other cell surface antigens are also present. For this purpose, A431 cells were used; these are human epidermal carcinoma cells with high levels of EGFR on their cell surface, and have been used previously to study EGFR targeting anti‐cancer drugs.19b, 24 In our on‐cell assay (Figure 2 A), 7D12 and its mutants were incubated with live A431 cells in a 96‐well plate, in media containing serum at 37 °C, thus allowing the binding to occur under physiologically relevant conditions. Following this, unbound 7D12 was removed, cells were fixed to the surface of the plate, and the bound 7D12 was assessed via its C‐terminus hexa‐histidine (His6) tag (Figure 2 A, and Page S5). Unlike several other techniques used for measuring protein‐protein interaction, this approach does not require sophisticated instrumentation or purified EGFR, and assesses the binding of antibody to EGFR on a cell surface. The technique is similar to whole cell enzyme‐linked immunosorbent assay (ELISA), and on‐cell western used for quantification of cell surface antigens.^25^ A series of control experiments were performed to demonstrate the viability of the on‐cell assay used in this study. When 7D12 was incubated with MDA‐MB‐231, a cell line with low levels of EGFR, often used as a negative control,^24^ the chemiluminescence signal was significantly lower compared to the signal for A431 cancer cell line (Figure S6). This supports our premise that the observed chemiluminescence is due to specific interaction between 7D12 and EGFR, and not due to non‐specific binding of 7D12 to the cell surface. We also measured the binding of an unrelated His6‐tagged antibody fragment, RR6‐VHH,^26^ to A431 cells using this assay. Near‐background level of chemiluminescence was observed with RR6‐VHH, demonstrating that the observed signal is not due to the non‐specific interaction of antibody fragments with A431 cancer cells (Figure S7). Prior to measuring the binding of photocaged mutants of 7D12, we used ESI‐MS to confirm light‐mediated decaging of 7D12**pcY**32, 7D12**pcY**109, and 7D12**pcY**113. The molecular weight of all the **pcY** mutants was reduced to that of wt7D12 after irradiation with 365 nm light for 4 min, confirming the loss of *o*‐nitrobenzyl group from tyrosine residues in the photocaged mutants (Figure 2 B).

On performing the on‐cell assay, it was observed that for wt7D12, as the concentration was increased from 0 to 100 nm, an increase in chemiluminescence occurs, followed by saturation of signal at higher concentration (Figure 2 C). For the 7D12**pcY**32 and 7D12**pcY**113 mutants, near‐background chemiluminescence signal was observed even up to 500 nm concentration (Figure 2 C and Figure S8), demonstrating that 7D12‐EGFR binding is inhibited due to the presence of **pcY** at positions 32 and 113 in 7D12. Interestingly, **pcY** at position 109, despite being at the binding interface, does not inhibit 7D12‐EGFR binding. We explain this difference in binding behaviour of mutants using computational simulations later in this study. Upon irradiating with 365 nm light (Page S5), 7D12**pcY**32 and 7D12**pcY**113 exhibit chemiluminescence signals similar to wt7D12, consistent with 7D12‐EGFR binding. This experiment demonstrates light‐mediated activation of antibody‐antigen binding in 7D12 mutants (Figure 2 C).

We estimated the binding affinity of 7D12 to EGFR on the surface of A431 cells from the chemiluminescence signal measured as a function of the concentration of 7D12 (Figure 2 D and Figure S9). The *K*_D_ for wt7D12 and 7D12**pcY**109 before irradiation with 365 nm light were estimated to be 23(±2.6) nm and 31(±1.5) nm, respectively. The *K*_D_ for wt7D12 calculated here is in close agreement with the value reported in a previous study.19a For caged 7D12**pcY**32 and 7D12**pcY**113, the *K*_D_ is expected to be greater than 500 nm, as near‐background chemiluminescence was observed till 500 nm concentration. After irradiation with 365 nm light, the *K*_D_ for wt7D12, and decaged 7D12**pcY**32, 7D12**pcY**109, and 7D12**pcY**113, were estimated to be 20(±1.8) nm, 37(±2.6) nm, 27(±1.6) nm and 38(±2.6) nm, respectively. For decaged 7D12**pcY**32 and 7D12**pcY**113, the chemiluminescence intensity at saturation is 86 % and 76 %, respectively, of the intensity for wt7D12; indicating that binding is recovered up to these levels (Figure S9). Accordingly, the *K*_D_ for decaged 7D12**pcY**32 and 7D12**pcY**113 are higher than for wt7D12.

For such photocaged antibodies to be useful in the clinic, the effect of 365 nm light as a cause for cell toxicity needs to be examined. Hence, we performed experiments to assess the viability of A431 cells after irradiation with 365 nm light for 4 min, which were the same conditions as for 7D12 decaging. The cells were then allowed to proliferate for 48 h, and cell viability was examined using the resazurin assay (Page S6 and Figure S10).^27^ The number of viable cells reduced by ≈12 % upon irradiation, leaving 88 % cells viable‐ demonstrating that radiation exposure conditions used for decaging 7D12 causes low toxicity in A431 cells.

**Molecular dynamics (MD) simulations explain the difference in effect of pcY on 7D12–EGFR interaction among mutants**. In the 7D12–EGFR domain III complex structure (PDB ID: 4KRL), Y32, Y109, and Y113 residues in 7D12 lie at the binding interface. Hence, in our experiments, we expected that substituting any of these tyrosine residues with **pcY** could inhibit or affect 7D12–EGFR binding. While two of the mutants show expected behavior, i.e., significantly reduced binding to EGFR, the third mutant does bind to EGFR. We investigated this difference in binding behaviour through a description of 7D12–EGFR interactions and dynamics in the presence and absence of photocaging group, using MD simulations.

All‐atom MD simulations were performed for four systems, starting from the 7D12–EGFR domain III crystal structure (PDB ID: 4KRL)^23^ for wt7D12, and with **pcY** substitutions in mutants, 7D12**pcY**32, 7D12**pcY**109, and 7D12**pcY**113, respectively, in the presence of explicit water and ions, using NAMD 2.12^28^ (Page S6).

**Figure 3:**
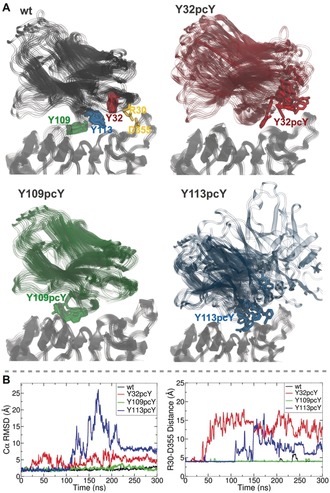
MD simulations of wt7D12 and its photocaged mutants show that wt7D12 and 7D12**pcY**109 form more stable complexes with EGFR domain III as compared to 7D12**pcY**32 and 7D12**pcY**113. A) Simulation snapshots taken at intervals of 30 ns during 300 ns simulations for each system, (EGFR: grey for all, wt7D12: black, 7D12**pcY**32: red, 7D12**pcY**109: green, 7D12**pcY**113: blue) highlight the extent of motion of 7D12. B) Left: RMSDs from starting structure for protein Cα atoms during simulations show large deviations for 7D12**pcY**32 and 7D12**pcY**113. Right: The R30–D355 salt‐bridge (residues shown in (A) wt7D12 snapshot, in yellow), monitored as the distance between the R30 guanidine C and the D355 carboxyl C, breaks frequently in the 7D12**pcY**32 and 7D12**pcY**113 systems. These observations suggest that the presence of **pcY** at positions 32 and 113 destabilizes the 7D12–EGFR domain III complex.

Comparing the dynamics of the systems during 300 ns simulations each, it is seen that wt7D12 and 7D12**pcY**109 remain bound to EGFR, while 7D12**pcY**32 and 7D12**pcY**113 show unbinding from EGFR for prolonged periods (Figure 3 A). The root mean square deviation (RMSD) of the complex from the starting conformation (Figure 3 B) shows a larger extent of movement in 7D12**pcY**32 and 7D12**pcY**113 compared to wt7D12 and 7D12**pcY**109. Visual analysis as well as measuring the number of contacts maintained by 7D12 with EGFR (Figure S11) confirms that this is due to frequent unbinding of 7D12 in the former two systems. Furthermore, a salt bridge formed by R30 in 7D12, and D355 in EGFR, described in previous experimental studies to play a key role in 7D12–EGFR binding,^23^ shows frequent breakage in 7D12**pcY**32 and 7D12**pcY**113, while remaining stable for wt7D12 and 7D12**pcY**109 (Figure 3 B).

Looking closely at the 7D12–EGFR interface, it is seen from the wt7D12–EGFR simulations that Y32 and Y113, both form some non‐specific interactions with EGFR, mainly with L325, and as such any notable hydrogen bonding or packing interactions are not seen. Upon substitution by **pcY**, in both cases, the additional *o*‐nitrobenzyl group protrudes into the binding interface, contacting several EGFR residues. Although initially appearing to be accommodated at the binding interface, as the simulation proceeds, it does not form stable contacts that could compensate for the crowding caused in the region, disrupting binding. On examining Y109, which is also at the binding interface, it is seen to remain oriented nearly parallel to the EGFR surface throughout the wt7D12 simulations, interacting significantly with only S418 on EGFR. The 7D12**pcY**109–EGFR complex simulation demonstrates that the additional *o*‐nitrobenzyl group is accommodated in a large solvent‐accessible cleft, not causing steric clashes, and allowing the complex to remain bound.

While providing an explanation for the different behavior of the Y109 mutant, this study also demonstrates the effectiveness of MD simulations to obtain details about inter‐residue interactions in proteins containing unnatural amino acids. The methodology and force field parameters developed here for **pcY** can also be utilized in future studies to design a predictive protocol to determine candidate residues for **pcY** substitution in other proteins.

**Light‐dependent delivery of fluorophores to the surface of live EGFR‐positive cancer cells**. We designed experiments to examine and provide evidence that photoactive antibodies can mediate light‐dependent delivery of small molecules to the surface of live A431 cells. wt7D12 and 7D12**pcY**32 were labeled using N‐hydroxysuccinimide (NHS) ester of a fluorophore, BODIPY‐TMR‐X (Page S6). We first assessed if the presence of this label on 7D12 influences 7D12‐EGFR binding, using our on‐cell assay. Comparison of unlabeled and labeled 7D12 reveal that the binding is reduced by ≈1.5‐fold due to the presence of the BODIPY‐TMR‐X label (Figure S12), hence at least 1.5‐fold higher concentration of labeled sample would be required for further experiments.

**Figure 4:**
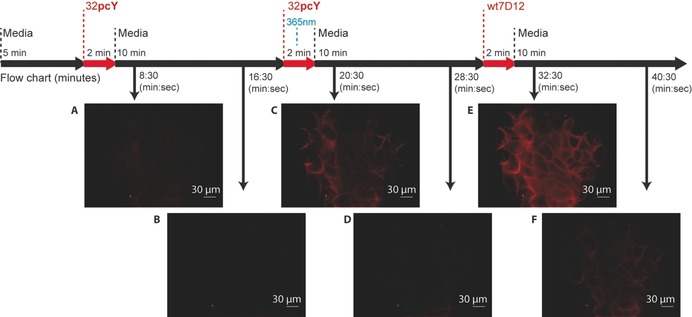
Light‐mediated delivery of fluorophores by photoactive antibodies on live A431 cells. A) Labeled 7D12**pcY**32 is injected at 5 min. Near‐background fluorescence is observed 1.5 min after passing labeled 7D12**pcY**32 over live A431 cells demonstrating that due to the presence of caged group, 7D12**pcY**32 does not bind to the cell surface. B) Background fluorescence before re‐injecting labeled 7D12**pcY**32. C) Labeled 7D12**pcY**32 was injected at 17 min and the irradiated with 365 nm light at 18 min (1 min after injecting 7D12**pcY**32) for 1 min. Significant fluorescence was observed 1.5 min after stopping the injection of labeled 7D12**pcY**32, demonstrating light‐dependent localisation of 7D12 on the surface of A431 cells. D) Fluorescence from 7D12 reduces to background level. E) Labeled wt7D12 was injected at 29 min. Significant fluorescence observed 1.5 min after stopping the injection of labeled wt7D12 due to localisation of labeled wt7D12 on the surface of A431 cells. F) Fluorescence from wt7D12 slowly reduces to near background level. See Movie S1 in the Supporting Information.

The light‐dependent localisation of photoactive antibody on the surface of live A431 cells was evaluated using fluorescence microscopy in a dynamic experimental setup. The microscope was fitted with a flow chamber, with the flow rate of media and the labeled antibody fragments fixed at 1 mL min^−1^ throughout the experiment. Images were acquired every 30 s, except during irradiation with 365 nm light. Media was initially passed for 5 min through the chamber containing A431 cells, followed by 500 nm solution of labeled 7D12**pcY**32 for 2 min, and then again media for 10 min. Fluorescence due to the passing fluorophore, is observed only during the passage of labeled 7D12**pcY**32; no signal attributable to localisation of fluorophore on the cell surface is seen (Supporting Movie S1). Next, labeled 7D12**pcY**32 was again passed through the chamber for 2 min, but this time, after 1 min, the chamber was irradiated with 365 nm light for 1 min for decaging **pcY**. These irradiation conditions were seen to exert low toxicity to A431 cells (Page S6 and Figure S13). Subsequent to the initial fluorescence due to the movement of labeled 7D12**pcY**32, localisation of fluorescence signal on the cell surface was observed even 2 min after stopping the injection of labeled 7D12**pcY**32. Comparison of fluorescence signals observed 1.5 min after stopping the flow of labeled 7D12**pcY**32 without (Figure 4 A), and with (Figure 4 C) irradiation, demonstrates light‐dependent delivery of fluorophores mediated by 7D12 to the surface of live A431 cells. The fluorescence signal from 7D12 bound to the surface of A431 cells eventually decays to background level, which could possibly be due to endocytosis of 7D12, or degradation of the fluorophore and/or photobleaching (Figure 4 D). Finally, as a control, 500 nm solution of labeled wt7D12 is passed through the chamber for 2 min. As expected, a strong fluorescence signal was observed over 1.5 mins after stopping the flow of labelled wt7D12, consistent with receptor‐mediated localisation of wt7D12 (Figure 4 E). The fluorescence signal for wt7D12 is stronger than for decaged 7D12**pcY**32 (Figure 4 C) presumably because the latter flows in the caged form for 1 min and decaged form for 1 min, thus reducing the effective concentration of actively binding 7D12, when compared to wt7D12 that flows for 2 min. Overall, these results are consistent with light‐dependent delivery of fluorophores on the surface of EGFR‐positive cancer cells.

This fluorescence microscopy study demonstrates that photoactive antibodies can deliver small molecules to the surface of specific cancer cells in a light‐dependent manner. Extending these antibodies to deliver cytotoxic drugs will provide a highly targeted, light‐mediated and receptor‐specific approach for cancer therapy.

The photoactive antibody fragments developed in this report have a molecular weight difference of less than 1 % from wild type, and hence expected to have similar pharmacokinetic properties. Introducing such modifications in currently used therapeutic antibodies could allow 365 nm UV light‐assisted targeting of cancer cells close to the skin surface. It has been shown that UV radiations can penetrate through murine skin to activate intradermally injected light‐sensitive therapeutics.^29^ UV light is also being used clinically in combination with the drug, psoralen, for treatment of skin cancer.^30^ LEDs emitting UV light are under investigation for treatment of Vitamin D deficiency,^31^ and, there have been investigations on activation of antibody‐based drugs using UV radiations, for treatment of ovarian cancer in mice models.10b The extension of the present work towards utilizing near‐infrared (IR) radiation instead of UV, is expected to widen its applicability as IR radiation penetrates deeper into tissue. The use of near‐IR light assisted by upconverting nanoparticles^32^ or two‐photons^33^ may also allow activation of UV‐sensitive photocaged antibodies in deeper tissues. Photoactive antibodies may also be useful for treatment of tumors in body parts where surgical implantation of biocompatible light emitting diodes (LEDs)^34^ is possible and photoactivation can be achieved using these LEDs.

## Conclusion

We report highly efficient genetic site‐specific incorporation of unnatural amino acids into the antibody fragment, 7D12, expressed in *E. coli*. Replacement of specific tyrosine residues with unnatural photocaged tyrosine in the antigen binding site of 7D12, resulted in development of photoactive antibodies. Light‐mediated binding of photoactive antibodies to their target, EGFR, was demonstrated using a robust and simple assay performed on the surface of cancer cells. Computational methods were used to study the dynamics of 7D12–EGFR interaction and explain the effect of the photocaging group when placed at different sites in the 7D12–EGFR binding interface. Finally, we show in a dynamic fluorescence microscopy experiment that photoactive antibodies can deliver small molecules to the surface of live cancer cells in a light‐dependent manner. This demonstration of the use of genetically encoded photocaged amino acids to control antibody binding, opens a new dimension in the field of controlled antibody‐antigen interactions, with the potential for widespread applications to biotherapeutics and nanotechnology.

## Conflict of interest

The authors declare no conflict of interest.

## Supporting information

As a service to our authors and readers, this journal provides supporting information supplied by the authors. Such materials are peer reviewed and may be re‐organized for online delivery, but are not copy‐edited or typeset. Technical support issues arising from supporting information (other than missing files) should be addressed to the authors.

Supplementary

Supplementary

## References

[1a] S. Kassem , T. van Leeuwen , A. S. Lubbe , M. R. Wilson , B. L. Feringa , D. A. Leigh , Chem. Soc. Rev. 2017, 46, 2592–2621; 28426052 10.1039/c7cs00245a

[1b] C. Cheng , J. F. Stoddart , ChemPhysChem 2016, 17, 1780–1793; 26833859 10.1002/cphc.201501155

[1c] S. Ranallo , C. Prevost-Tremblay , A. Idili , A. Vallee-Belisle , F. Ricci , Nat. Commun. 2017, 8, 15150; 28480878 10.1038/ncomms15150 PMC5424144

[1d] C. Chou , D. D. Young , A. Deiters , Angew. Chem. Int. Ed. 2009, 48, 5950–5953; 10.1002/anie.200901115 19569148

[2a] S. M. Douglas , I. Bachelet , G. M. Church , Science 2012, 335, 831–834; 22344439 10.1126/science.1214081

[2b] J. Broichhagen , M. Schonberger , S. C. Cork , J. A. Frank , P. Marchetti , M. Bugliani , A. M. Shapiro , S. Trapp , G. A. Rutter , D. J. Hodson , D. Trauner , Nat. Commun. 2014, 5, 5116; 25311795 10.1038/ncomms6116 PMC4208094

[2c] S. Erbas-Cakmak , D. A. Leigh , C. T. McTernan , A. L. Nussbaumer , Chem. Rev. 2015, 115, 10081–10206. 26346838 10.1021/acs.chemrev.5b00146 PMC4585175

[3a] Y. S. Chen , M. Y. Hong , G. S. Huang , Nat. Nanotechnol. 2012, 7, 197–203; 22367097 10.1038/nnano.2012.7

[3b] G. J. Weiner , Nat. Rev. Cancer 2015, 15, 361–370. 25998715 10.1038/nrc3930 PMC4491443

[4] L. R. Desnoyers , O. Vasiljeva , J. H. Richardson , A. Yang , E. E. Menendez , T. W. Liang , C. Wong , P. H. Bessette , K. Kamath , S. J. Moore , J. G. Sagert , D. R. Hostetter , F. Han , J. Gee , J. Flandez , K. Markham , M. Nguyen , M. Krimm , K. R. Wong , S. Liu , P. S. Daugherty , J. W. West , H. B. Lowman , Sci. Transl. Med. 2013, 5, 207ra144. 10.1126/scitranslmed.3006682 24132639

[5] S. B. Gunnoo , H. M. Finney , T. S. Baker , A. D. Lawson , D. C. Anthony , B. G. Davis , Nat. Commun. 2014, 5, 4388. 25073737 10.1038/ncomms5388 PMC4124856

[6a] B. S. Howerton , D. K. Heidary , E. C. Glazer , J. Am. Chem. Soc. 2012, 134, 8324–8327; 22553960 10.1021/ja3009677

[6b] F. Reessing , W. Szymanski , Curr. Med. Chem. 2017, 24, 4905–4950. 27601187 10.2174/0929867323666160906103223

[7] A. M. Scott , J. D. Wolchok , L. J. Old , Nat. Rev. Cancer 2012, 12, 278–287. 22437872 10.1038/nrc3236

[8] T. T. Hansel , H. Kropshofer , T. Singer , J. A. Mitchell , A. J. George , Nat. Rev. Drug Discovery 2010, 9, 325–338. 20305665 10.1038/nrd3003

[9] M. Mitsunaga , M. Ogawa , N. Kosaka , L. T. Rosenblum , P. L. Choyke , H. Kobayashi , Nat. Med. 2011, 17, 1685–1691. 22057348 10.1038/nm.2554 PMC3233641

[10a] C. H. Self , S. Thompson , Nat. Med. 1996, 2, 817–820; 8673931 10.1038/nm0796-817

[10b] S. Thompson , J. Dessi , C. H. Self , MAbs 2009, 1, 348–356. 20068406 10.4161/mabs.1.4.9045 PMC2726611

[11a] D. D. Young , P. G. Schultz , ACS Chem. Biol. 2018, 13, 854–870; 29345901 10.1021/acschembio.7b00974 PMC6061972

[11b] J. W. Chin , Annu. Rev. Biochem. 2014, 83, 379–408; 24555827 10.1146/annurev-biochem-060713-035737

[11c] A. Dumas , L. Lercher , C. D. Spicer , B. G. Davis , Chem. Sci. 2015, 6, 50–69; 28553457 10.1039/c4sc01534g PMC5424465

[11d] L. Seidel , I. Coin , Methods Mol. Biol. 2018, 1728, 221–235. 29405001 10.1007/978-1-4939-7574-7_14

[12] J. Hemphill , C. Chou , J. W. Chin , A. Deiters , J. Am. Chem. Soc. 2013, 135, 13433–13439. 23931657 10.1021/ja4051026 PMC4188981

[13] A. Gautier , A. Deiters , J. W. Chin , J. Am. Chem. Soc. 2011, 133, 2124–2127. 21271704 10.1021/ja1109979 PMC3048767

[14] D. P. Nguyen , M. Mahesh , S. J. Elsasser , S. M. Hancock , C. Uttamapinant , J. W. Chin , J. Am. Chem. Soc. 2014, 136, 2240–2243. 24479649 10.1021/ja412191m PMC4333589

[15] J. K. Böcker , W. Dörner , H. D. Mootz , Chem. Commun. 2019, 55, 1287–1290. 10.1039/c8cc09204d 30633261

[16a] N. Normanno , A. De Luca , C. Bianco , L. Strizzi , M. Mancino , M. R. Maiello , A. Carotenuto , G. De Feo , F. Caponigro , D. S. Salomon , Gene 2006, 366, 2–16; 16377102 10.1016/j.gene.2005.10.018

[16b] S. Li , K. R. Schmitz , P. D. Jeffrey , J. J. Wiltzius , P. Kussie , K. M. Ferguson , Cancer Cell 2005, 7, 301–311. 15837620 10.1016/j.ccr.2005.03.003

[17a] N. E. Hynes , H. A. Lane , Nat. Rev. Cancer 2005, 5, 341–354; 15864276 10.1038/nrc1609

[17b] R. Peréz-Soler , L. Saltz , J. Clin. Oncol. 2005, 23, 5235–5246. 16051966 10.1200/JCO.2005.00.6916

[18] S. Muyldermans , Annu. Rev. Biochem. 2013, 82, 775–797. 23495938 10.1146/annurev-biochem-063011-092449

[19a] R. C. Roovers , T. Laeremans , L. Huang , S. De Taeye , A. J. Verkleij , H. Revets , H. J. de Haard , P. M. van Bergen en Henegouwen , Cancer Immunol. Immunother. 2007, 56, 303–317; 16738850 10.1007/s00262-006-0180-4 PMC11030579

[19b] R. C. Roovers , M. J. Vosjan , T. Laeremans , R. el Khoulati , R. C. de Bruin , K. M. Ferguson , A. J. Verkleij , G. A. van Dongen , P. M. van Bergen en Henegouwen , Int. J. Cancer 2011, 129, 2013–2024. 21520037 10.1002/ijc.26145 PMC4197845

[20a] G. Biffi , D. Tannahill , J. McCafferty , S. Balasubramanian , Nat. Chem. 2013, 5, 182–186; 23422559 10.1038/nchem.1548 PMC3622242

[20b] C. D. Martin , G. Rojas , J. N. Mitchell , K. J. Vincent , J. Wu , J. McCafferty , D. J. Schofield , BMC Biotechnol. 2006, 6, 46. 17156422 10.1186/1472-6750-6-46 PMC1712229

[21a] A. Deiters , D. Groff , Y. Ryu , J. Xie , P. G. Schultz , Angew. Chem. Int. Ed. 2006, 45, 2728–2731; 10.1002/anie.200600264 16548032

[21b] E. Arbely , J. Torres-Kolbus , A. Deiters , J. W. Chin , J. Am. Chem. Soc. 2012, 134, 11912–11915. 22758385 10.1021/ja3046958

[22a] A. Chatterjee , S. B. Sun , J. L. Furman , H. Xiao , P. G. Schultz , Biochemistry 2013, 52, 1828–1837; 23379331 10.1021/bi4000244 PMC3855549

[22b] A. Sachdeva , K. Wang , T. Elliott , J. W. Chin , J. Am. Chem. Soc. 2014, 136, 7785–7788. 24857040 10.1021/ja4129789 PMC4333588

[23] K. R. Schmitz , A. Bagchi , R. C. Roovers , P. M. van Bergen en Henegouwen , K. M. Ferguson , Structure 2013, 21, 1214–1224. 23791944 10.1016/j.str.2013.05.008 PMC3733345

[24] R. Railkar , L. S. Krane , Q. Q. Li , T. Sanford , M. R. Siddiqui , D. Haines , S. Vourganti , S. J. Brancato , P. L. Choyke , H. Kobayashi , P. K. Agarwal , Mol. Cancer Ther. 2017, 16, 2201–2214. 28619755 10.1158/1535-7163.MCT-16-0924 PMC5628127

[25a] W. Jiang , S. Cossey , J. N. Rosenberg , G. A. Oyler , B. J. Olson , D. P. Weeks , BMC Plant Biol. 2014, 14, 244; 25252698 10.1186/s12870-014-0244-0 PMC4181299

[25b] B. P. Delisle , H. A. Underkofler , B. M. Moungey , J. K. Slind , J. A. Kilby , J. M. Best , J. D. Foell , R. C. Balijepalli , T. J. Kamp , C. T. January , J. Biol. Chem. 2009, 284, 2844–2853. 19029296 10.1074/jbc.M807289200 PMC2631954

[26] S. Spinelli , L. G. Frenken , P. Hermans , T. Verrips , K. Brown , M. Tegoni , C. Cambillau , Biochemistry 2000, 39, 1217–1222. 10684599 10.1021/bi991830w

[27] J. O'Brien , I. Wilson , T. Orton , F. Pognan , Eur. J. Biochem. 2000, 267, 5421–5426. 10951200 10.1046/j.1432-1327.2000.01606.x

[28] J. C. Phillips , R. Braun , W. Wang , J. Gumbart , E. Tajkhorshid , E. Villa , C. Chipot , R. D. Skeel , L. Kale , K. Schulten , J. Comput. Chem. 2005, 26, 1781–1802. 16222654 10.1002/jcc.20289 PMC2486339

[29] T. Lucas , F. Schafer , P. Muller , S. A. Eming , A. Heckel , S. Dimmeler , Nat. Commun. 2017, 8, 15162. 28462946 10.1038/ncomms15162 PMC5418571

[30a] R. Edelson , C. Berger , F. Gasparro , B. Jegasothy , P. Heald , B. Wintroub , E. Vonderheid , R. Knobler , K. Wolff , G. Plewig , et al., N. Engl. J. Med. 1987, 316, 297–303; 3543674 10.1056/NEJM198702053160603

[30b] H. T. Greinix , B. Volc-Platzer , W. Rabitsch , B. Gmeinhart , C. Guevara-Pineda , P. Kalhs , J. Krutmann , H. Honigsmann , M. Ciovica , R. M. Knobler , Blood 1998, 92, 3098–3104. 9787144

[31] T. A. Kalajian , A. Aldoukhi , A. J. Veronikis , K. Persons , M. F. Holick , Sci. Rep. 2017, 7, 11489. 28904394 10.1038/s41598-017-11362-2 PMC5597604

[32] S. Wen , J. Zhou , K. Zheng , A. Bednarkiewicz , X. Liu , D. Jin , Nat. Commun. 2018, 9, 2415. 29925838 10.1038/s41467-018-04813-5 PMC6010470

[33] J. Croissant , M. Maynadier , A. Gallud , H. Peindy N'dongo , J. L. Nyalosaso , G. Derrien , C. Charnay , J. O. Durand , L. Raehm , F. Serein-Spirau , N. Cheminet , T. Jarrosson , O. Mongin , M. Blanchard-Desce , M. Gary-Bobo , M. Garcia , J. Lu , F. Tamanoi , D. Tarn , T. M. Guardado-Alvarez , J. I. Zink , Angew. Chem. Int. Ed. 2013, 52, 13813–13817; 10.1002/anie.201308647 PMC3940420 24214916

[34] H. Zhang , J. A. Rogers , Adv. Opt. Mater. 2019, 7, 1800936–1800936.

