# Supplementary material for: Site‐Specific Encoding of Photoactivity in Antibodies Enables Light‐Mediated Antibody–Antigen Binding on Live Cells
Source: Angew Chem Int Ed Engl. 2019 Oct 31;58(50):17986–93. doi: 10.1002/anie.201908655 (PMC6973043; doi:10.1002/anie.201908655)
Supplement: Supplementary file 1 — Supplementary [file ANIE-58-17986-s001.pdf]

Angewandte Chemie
Eine Zeitschrift der Gesellschaft Deutscher Chemiker

Supporting Information

Site-Specific Encoding of Photoactivity in Antibodies Enables Light-Mediated Antibody–Antigen Binding on Live Cells

*Thomas Bridge, Saher A. Shaikh, Paul Thomas, Joaquin Botta, Peter J. McCormick, and Amit Sachdeva**

anie_201908655_sm_miscellaneous_information.pdf
anie_201908655_sm_Movie_S1.qt

Author Contributions

T.B. Data curation: Lead; Formal analysis: Lead; Methodology: Lead; Writing—Review & Editing: Supporting; Performed all primary lab experiments and data analysis. : Lead
S.S. Data curation: Lead; Formal analysis: Lead; Methodology: Lead; Software: Lead; Validation: Lead; Writing—Original Draft: Lead; Writing—Review & Editing: Lead; Performed and wrote the sections on computational studies. : Lead
P.T. Data curation: Supporting; Software: Supporting; Visualization: Supporting; Provided training and support for microscopy experiments.: Supporting
J.B. Provided initial training in on-cell experiments: Supporting
P.M. Provided A431 cells and initial assistance in on-cell experiments: Supporting
A.S. Conceptualization: Lead; Data curation: Lead; Formal analysis: Lead; Funding acquisition: Lead; Investigation: Lead; Methodology: Lead; Project administration: Lead; Resources: Lead; Supervision: Lead; Validation: Lead; Writing—Original Draft: Lead; Writing—Review & Editing: Lead.

Table of Contents

Cell lines and unnatural amino acids .... page S3
Construction of pSANG10_7D12 .... page S3
Construction of pSANG10_7D12-32TAG, pSANG10_7D12-109TAG, and pSANG10_7D12-113TAG plasmids .... page S3
Construction of pULTRA_*Mj*pcYRS/*Mj*tRNA$_{CUA}$ plasmid .... page S3
Construction of pULTRA_PylRS/PyltRNA$_{CUA}$ plasmid .... page S3
Expression of wt7D12 .... page S3
Screening of suppressor plasmids pSUP_MjCNFRS/6xMjtRNACUA, pEvolv_2xMjCNFRS/MjtRNACUA, and pULTRA_*Mj*CNFRS/*Mj*tRNA$_{CUA}$ for site-specific incorporation of 4-Azido-L-phenylalanine (AzF) .... page S4
Screening of suppressor plasmids pCDF_3xPylRS/PyltRNA$_{CUA}$, and pULTRA_PylRS/PyltRNA$_{CUA}$ for site-specific incorporation of N6-(tert-butoxycarbonyl)-L-lysine (BocK) and N6-[(2-propynyloxy)carbonyl]-L-lysine (CAK) .... page S4
Expression and purification of amber mutants of 7D12 with site-specifically incorporated photocaged tyrosine (**pcY**) .... page S5
Conditions for light-mediated decaging of photoactive antibodies in on-cell assay .... page S5
Cell viability experiments under irradiation conditions for on-cell assay .... page S6
On-cell assay for measuring the binding of His-tagged antibody fragments .... page S6
Computational methods .... page S6
Labelling of antibody fragments with BODIPY-TMR-X .... page S6
Microscopy set-up .... page S6
Conditions for light-mediated decaging of photoactive antibodies in microscopy experiments .... page S5
Cell viability experiments under irradiation conditions for microscopy experiments .... page S6
Supporting figures .... page S7
References .... page S20

Experimental Procedures

Cell lines and unnatural amino acids: Epithelial squamous carcinoma cell line, A431, and human breast adenocarcinoma cell line, MDA-MB-231, were purchased from Sigma-Aldrich. All mammalian cell lines were cultured in DMEM (Gibco, Invitrogen) containing L-glutamine, 4.5 g/L D-Glucose, 110 mg/L Sodium pyruvate, 10% (v/v) foetal bovine serum (FBS), and 1% (v/v) cocktail of penicillin and streptomycin (PEN/STREP, Sigma-Aldrich). This medium will be referred to as “complete medium”. Mammalian cells were grown in humidified atmosphere in 5% CO_2 at 37°C. p-Azido-L-phenylalanine and N6-(tert-butoxycarbonyl)-L-lysine were purchased from BACHEM. O-(2-Nitrobenzyl)-L-tyrosine (photocaged tyrosine, **pcY**) and N6-[(2-propynyloxy)carbonyl]-L-lysine were synthesized using procedures similar to reported earlier.[1]

Construction of pSANG10_7D12: pSANG10-3F-BG4 (Addgene plasmid # 55756) has been earlier employed for periplasmic expression of single chain antibody fragment (scFv), BG4.[2] pSANG10-3F-BG4 was digested with NdeI and HindIII to remove the coding DNA sequence (CDS) for BG4 fragment. After digestion, the reaction mixture was run on 1% agarose gel. The band corresponding to pSANG10 backbone was excised and extracted using QIAquick Gel Extraction Kit (QIAGEN). The codon optimised CDS for 7D12 (procured as gene fragment from Integrated DNA Technologies (IDT), Figure S1) was subsequently cloned into the pSANG10 backbone using Gibson cloning (New England Biolabs). After cloning the sequence of pSANG10_7D12 was confirmed by Sanger Sequencing.

Construction of pSANG10_7D12-32TAG, pSANG10_7D12-109TAG, and pSANG10_7D12-113TAG plasmids: For construction of pSANG10_7D12-32TAG mutant, the pSANG10_7D12 plasmid was digested with XbaI and SalI. For construction of pSANG10_7D12-109TAG and pSANG10_7D12-113TAG mutants, the pSANG10_7D12 plasmid was digested with SalI and HindIII. After digestion, the plasmid backbone was purified on 1% agarose and the DNA extracted using QIAquick Gel Extraction Kit (QIAGEN). The mutations in 7D12 were introduced by cloning short gene fragments (IDT) with the corresponding mutations into the digested plasmids. Gibson cloning (NEB) was used for this purpose. After cloning the sequence of mutated plasmids was confirmed by Sanger Sequencing.

Construction of pULTRA_*Mj*pcYRS/*Mj*tRNA$_{CUA}$ plasmid: *Mj*TyrRS has been earlier evolved to genetically encode photocaged tyrosine (**pcY**) into proteins.[1a] The mutant *Mj*TyrRS protein for incorporating **pcY** has the following five mutations: Tyr32 → Gly32, Leu65 → Gly65, Phe108 → Glu108, Asp158 → Ser158 and Leu162 → Glu162. To construct pULTRA_*Mj*pcYRS/*Mj*tRNA$_{CUA}$, pULTRA_*Mj*CNFRS/*Mj*tRNA$_{CUA}$ plasmid (Addgene plasmid # 48215) was digested with NotI (New England Biolabs). After digestion, the reaction mixture was run on 1% agarose gel. The band corresponding to pULTRA_*Mj*tRNA$_{CUA}$ backbone (without aaRS) was excised and extracted using QIAquick Gel Extraction Kit (QIAGEN). The codon optimised CDS for *Mj*pcYRS (procured as gene fragment from Integrated DNA Technologies (IDT)) was subsequently cloned into the pULTRA_*Mj*tRNA$_{CUA}$ backbone using Gibson cloning (New England Biolabs). After cloning the sequence of pULTRA_*Mj*pcYRS/*Mj*tRNA$_{CUA}$ was confirmed by Sanger Sequencing.

Construction of pULTRA_PylRS/PyltRNA$_{CUA}$ plasmid: In order to construct pULTRA_PylRS/PyltRNA$_{CUA}$ plasmid, pULTRA_*Mj*CNFRS/*Mj*tRNA$_{CUA}$ plasmid was digested with PstI and XhoI (New England Biolabs). After digestion, the reaction mixture was run on 1% agarose gel. The band corresponding to pULTRA_*Mj*CNFRS backbone (without tRNA and flanking region) was excised and extracted using QIAquick Gel Extraction Kit (QIAGEN). The PyltRNA$_{CUA}$ having the same promoter and terminator as for *Mj*tRNA$_{CUA}$ (procured as gene fragment from Integrated DNA Technologies (IDT)) was subsequently cloned into the pULTRA_*Mj*CNFRS backbone using Gibson cloning (New England Biolabs). After cloning the sequence of pULTRA_*Mj*CNFRS/PyltRNA$_{CUA}$ was confirmed by Sanger Sequencing. In order to replace *Mj*CNFRS in pULTRA_*Mj*CNFRS/PyltRNA$_{CUA}$, the plasmid was digested with NotI (New England Biolabs). PylRS was PCR amplified from another high copy plasmid. PCR product and the digested plasmid were run on 1% agarose gel, the corresponding bands excised and DNA extracted using QIAquick Gel Extraction Kit (QIAGEN). PylRS (PCR product) was cloned into the pULTRA_ PyltRNA$_{CUA}$ backbone using Gibson cloning (New England Biolabs). After cloning the sequence of pULTRA_PylRS/PyltRNA$_{CUA}$ was confirmed by Sanger Sequencing.

Expression of wt7D12: For periplasmic expression of wt7D12, pSANG10_7D12 plasmid was transformed into BL21(DE3)pLysS chemically competent cells. After transformation, cells were recovered in 1 ml SOB medium for one hour at 37°C. 50 µl of recovered cells were transferred onto LB-agar plates supplemented with 50 µg ml^{-1} kanamycin. The plates were incubated overnight (37°C, 16 h). A single colony from this plate was used to inoculate 10 ml of 2xTY-GK media (2xTY media with 4% glucose, 50 µg ml^{-1} kanamycin) and incubated overnight (37°C, 220rpm, 16 h). Next day, this culture was used to subculture 500 ml 2xTY-GK, such that OD600= 0.1. This was then incubated until OD600 reached 0.4-0.6 (37°C, 220rpm, 2-3 h). IPTG (1 mM final concentration) was added to induce the expression of 7D12 and the culture was incubated overnight (30°C, 160rpm, 16 h). Next day, cells were pelleted (3,200g, 4°C, 10 minutes). The supernatant was discarded and the cells were resuspended in 25 ml periplasmic extraction buffer-1 (20% sucrose, 100 mM Tris-HCl, 1 mM EDTA, pH 8.0). The resuspended cells were incubated on ice for 30 minutes and then centrifuged (10,000g, 4°C, 10 minutes). The supernatant was removed and stored at 4°C- this is “periplasmic fraction-1”. The resulting pellet was resuspended in 25 ml periplasmic extraction buffer-2 (5 mM $MgCl_2$) and incubated on ice for 20 minutes. The samples were centrifuged (10,000g, 4°C, 10 minutes) and the supernatant was collected- this is “periplasmic fraction-2”. Both the periplasmic fractions were combined, passed through a 0.2 µm filter, and dialyzed overnight at 4°C against 1x phosphate buffered saline (PBS). For purification of 7D12 antibody fragment from the periplasmic mixture, 1 ml of Ni-NTA resin was added to the dialyzed fractions and mixed gently on a rocker (4°C, 1 h). This was transferred into a gravity-flow column and washed with 10 ml PBS buffer. Resin was then washed twice with 8 ml Ni-NTA wash buffer (50 mM Tris-HCl, 300 mM NaCl and 20 mM imidazole, pH

8.0). To elute the bound 7D12, 500 µL Ni-NTA elution buffer (50 mM Tris-HCl, 300 mM NaCl and 200 mM imidazole, pH 8.0) was added and incubated for 15 minutes at room temperature, this was repeated 8 times. Elution fractions were pooled and dialyzed overnight at 4°C against 1xPBS buffer. Dialyzed fractions were then concentrated by Vivaspin 500 columns with 3 kDa molecular weight cut-off (GE Healthcare) and the yields were determined using a calorimetric Pierce BCA protein assay (ThermoFisher Scientific) measured at 562 nm. The samples were subsequently analysed by SDS-PAGE. Nu-PAGE LDS loading buffer (Invitrogen) was added to samples and heated to 95°C for 15 minutes, then centrifuged (13,000 g, 15 minutes, 4°C) and loaded on a 4-12% Bis-Tris gel (Invitrogen) along with BIO-RAD pre-stained protein ladder (Precision Plus All Blue Protein Standards) as a marker. The gel was then stained with Coomassie Blue (InstantBlue, Expedeon) (Figure S2a) and the identity of the protein was further confirmed by electrospray ionization mass spectrometry coupled with liquid chromatography, LC-ESI-MS (Figure S2b). The molecular weights observed on SDS-PAGE gel and mass spectrometry were in good agreement with expected molecular weight of 7D12.

Screening of suppressor plasmids pSUP_*Mj*CNFRS/6xMjtRNA$_{CUA}$, pEvolv_2x*Mj*CNFRS/MjtRNA$_{CUA}$, and pULTRA_*Mj*CNFRS/*Mj*tRNA$_{CUA}$ for site-specific incorporation of 4-Azido-L-phenylalanine (AzF): Several suppressor plasmids containing aminoacyl-tRNA synthetase (aaRS)/tRNA pairs have been used for genetic encoding of unnatural amino acids in proteins. Here, we investigated the efficiency of three suppressor plasmids to site-specifically incorporate 4-Azido-L-phenylalanine (AzF) into 7D12. First, chemically competent BL21(DE3)pLysS cells containing pSANG10_7D12-32TAG were prepared. These cells were transformed with either pSUP_*Mj*CNFRS/6x*Mj*tRNA$_{CUA}$,[3] pEvolv_2x*Mj*CNFRS/*Mj*tRNA$_{CUA}$,[4] or pULTRA_*Mj*CNFRS/*Mj*tRNA$_{CUA}$[5] plasmids for incorporation of 4-azido-L-phenylalanine at position 32 in 7D12. The transformed cells were recovered in 1 ml SOB medium for one hour at 37°C. Recovered cells (50 µl) containing pSUP_*Mj*CNFRS/6x*Mj*tRNA$_{CUA}$ + pSANG10_7D12-32TAG, and pEvolv_2x*Mj*CNFRS/*Mj*tRNA$_{CUA}$ + pSANG10_7D12-32TAG were platted on LB-agar plates supplemented with 25 µg ml^{-1} chloramphenicol and 50 µg ml^{-1} kanamycin. Recovered cells (50 µl) containing pULTRA_*Mj*CNFRS/*Mj*tRNA$_{CUA}$ + pSANG10_7D12-32TAG were platted on LB-agar plates supplemented with 75 µg ml^{-1} spectinomycin and 50 µg ml^{-1} kanamycin. LB agar plates were incubated overnight (37°C, 16 h). A single colony from each plate was used for inoculation of 5 ml of either 2xTY-GKC media (2xTY media with 4% glucose, 50 µg ml^{-1} kanamycin, 25 µg ml^{-1} chloramphenicol) or 2xTY-GKS media (2xTY media with 4% glucose, 50 µg ml^{-1} kanamycin, 75 µg ml^{-1} spectinomycin), depending upon the antibiotic resistance marker on the suppressor plasmids. The cultures were incubated overnight (37°C, 220rpm, 16 h). Next day, OD600 for the overnight culture was measured and used to subculture 5 ml 2xTY-GKS or 2xTY-GKS media such that OD600= 0.1. This was then incubated until OD600 reached 0.4-0.6 (37°C, 220rpm, 2-3 h). All samples were induced with IPTG (1mM). The positive samples were also supplemented with 4mM 4-Azido-L-phenylalanine (AzF). The cultures were then incubated overnight (30°C, 160rpm, 16 h). Next day, 1 ml of cells were pelleted (3,200g, 4°C, 10 minutes). Supernatant was discarded and the cells were resuspended in 50 µl periplasmic extraction buffer-1 (20% sucrose, 100 mM Tris-HCl, 1 mM EDTA, pH 8.0). The resuspended cells were incubated on ice for 30 minutes and then centrifuged (10,000g, 4°C, 10 minutes). The supernatant was removed and stored at 4°C- this is “periplasmic fraction-1”. The resulting pellet was resuspended in 50 µl periplasmic extraction buffer-2 (5 mM $MgCl_2$) and incubated on ice for 20 minutes. The samples were centrifuged (10,000g, 4°C, 10 minutes) and the supernatant was collected- this is “periplasmic fraction-2”. Both the periplasmic fractions were combined, and passed through a 0.2-µm filter. 20 µl of periplasmic fraction was used for analysis using western blot. Nu-PAGE LDS loading buffer (Invitrogen) was added to the samples (20 µl), heated at 95°C for 15 minutes, centrifuged (13,000 g, 15 minutes, 4°C) and loaded on a 4-12% Bis-Tris gel (Invitrogen) along with BIO-RAD pre-stained protein ladder (Precision Plus All Blue Protein Standards) as a marker. After running the gel, the proteins in the gel were transferred to a nitrocellulose membrane using iBLOT 2 (Invitrogen). After transfer, the membrane was transferred in blocking buffer (10% Milk, PBS-Tween 0.1%) and incubated for 1 h (gentle rocking, room temperature). After removing blocking buffer, the membrane was incubated with primary antibody (Mouse-anti-6X-HIS tag, Invitrogen) at 1:1000 dilution in a solution of 1% milk in PBST (PBS-Tween 0.1%) (1h, room temperature, gentle rocking). After incubation with primary antibody, the membrane was washed with PBST (5 minutes, gentle rocking). The washing step was repeated three times. Subsequently, the membrane was incubated with secondary antibody (Anti-mouse, IgG, HPR-linked) at 1:3000 dilution in a solution of 1% milk in PBST (PBS-Tween 0.1%) (1h at room temperature or overnight at 4°C, gentle rocking). After incubation with secondary antibody, the membrane was washed with PBST (5 minutes, gentle rocking). The washing step was repeated three times. The membrane was developed using SuperSignal Chemiluminescent Substrate and imaged using BIORAD GelDoc XR+ (Figure S3).

Screening of suppressor plasmids pCDF_3xPylRS/PyltRNA$_{CUA}$, and pULTRA_PylRS/PyltRNA$_{CUA}$ for site-specific incorporation of N6-(tert-butoxycarbonyl)-L-lysine (BocK) and N6-[(2-propynyloxy)carbonyl]-L-lysine (CAK): We investigated the efficiency of two suppressor plasmids to site-specifically incorporate N6-(tert-butoxycarbonyl)-L-lysine (BocK) and N6-[(2-propynyloxy)carbonyl]-L-lysine (CAK) into 7D12. Chemically competent BL21(DE3)pLysS cells containing pSANG10_7D12-32TAG were transformed with either pCDF_3xPylRS/PyltRNA$_{CUA}$, or pULTRA_PylRS/PyltRNA$_{CUA}$ plasmids for incorporation of BocK and CAK at position 32 in 7D12. The transformed cells were recovered in 1 ml SOB medium for one hour at 37°C. Recovered cells (50 µl) containing pCDF_3xPylRS/PyltRNA$_{CUA}$ + pSANG10_7D12-32TAG, and pULTRA_PylRS/PyltRNA$_{CUA}$ + pSANG10_7D12-32TAG were platted on LB-agar plates supplemented with 75 µg ml^{-1} spectinomycin and 50 µg ml^{-1} kanamycin. LB agar plates were incubated overnight (37°C, 16 h). A single colony from each plate was used for inoculation of 50 ml of 2xTY-GKS media (2xTY media with 4% glucose, 50 µg ml^{-1} kanamycin, 75 µg ml^{-1} spectinomycin). The cultures were incubated overnight (37°C, 220rpm, 16 h). Next day, OD600 for the overnight culture was measured and used to subculture 50 ml 2xTY-GKS media such that OD600= 0.1. This was then incubated until OD600 reached 0.4-0.6 (37°C, 220rpm, 2-3 h). All samples were induced with IPTG (1mM). For each experiment, three cultures were prepared: a) without unnatural amino acid, b) with 8 mM of BocK, and c) with 8 mM of CAK. These cultures were then incubated overnight (30°C, 160rpm, 16 h). Next day, 50 ml of cells were pelleted (3,200g, 4°C, 10 minutes). Supernatant was discarded and the cells were resuspended in 2.5 ml of periplasmic extraction buffer-1 (20% sucrose, 100 mM Tris-HCl, 1 mM EDTA, pH 8.0). The resuspended cells were incubated on ice for 30 minutes and then centrifuged (10,000g, 4°C, 10 minutes). The supernatant was removed and stored at 4°C- this is “periplasmic fraction-1”. The resulting pellet was resuspended in 2.5 ml periplasmic extraction buffer-2 (5 mM $MgCl_2$) and incubated on ice for 20 minutes. The samples were centrifuged (10,000g, 4°C, 10 minutes) and the supernatant was collected- this is “periplasmic fraction-2”. Both the periplasmic fractions were combined, and passed through a 0.2-µm filter, and dialyzed overnight at 4°C against 1x phosphate buffered saline (PBS). 100 µL of Ni-NTA resin was added to the dialyzed solution and mixed gently on a rocker (4°C, 1 h). The mixture was centrifuged at 300g for 1 min to collect the resin at the bottom of the tube and the supernatant was discarded. The resin with the bound protein was washed 5 times with 1 ml of 1xPBS and subsequently, 5 times with 1 ml Ni-NTA wash buffer (50 mM Tris-HCl, 300 mM NaCl and 20 mM imidazole, pH 8.0);

each time 1 ml of 1xPBS or Ni-NTA wash buffer was added to the resin, mixed thoroughly, centrifuged at 300g for 1 min and the supernatant was discarded. After washing, 100µl of Nu-PAGE LDS loading buffer (Invitrogen) was added to the resin, heated at 95°C for 15 minutes, centrifuged (13,000g, 15 minutes, 4°C) and 20 µl of supernatant loaded on a 4-12% Bis-Tris gel (Invitrogen) along with BIO-RAD pre-stained protein ladder (Precision Plus All Blue Protein Standards) as a marker. The gel was then stained with Coomassie Blue (InstantBlue, Expedeon) (Figure S4).

Expression and purification of amber mutants of 7D12 with site-specifically incorporated photocaged tyrosine (pcY): Chemically competent BL21(DE3)pLysS cells containing pULTRA_*Mj*pcYRS/*Mj*tRNA$_{CUA}$ were transformed with pSANG10_7D12, pSANG10_7D12-32TAG, pSANG10_7D12-109TAG, or pSANG10_7D12-113TAG plasmids. After transformation, cells were recovered in 1 ml SOB medium for one hour at 37°C. 50 µl of recovered cells were transferred onto LB-agar plates supplemented with 50 µg ml^{-1} kanamycin and 75 µg ml^{-1} spectinomycin. The plates were incubated overnight (37°C, 16 h). A single colony from each plate was used to inoculate 50 ml of 2xTY-GKS media (2xTY media with 4% glucose, 50 µg ml^{-1} kanamycin and 75 µg ml^{-1} spectinomycin) and incubated overnight (37°C, 220rpm, 16 h). Next day, this culture was used to subculture 500 ml 2xTY-GKS media, such that OD600= 0.1. This was then incubated until OD600 reached 0.4-0.6 (37°C, 220rpm, 2-3 h). IPTG (1 mM final concentration) was added to induce the expression of 7D12 and the culture was supplemented with 4 mM photocaged tyrosine (**pcY**) for expression with unnatural amino acid (positive samples in Figure 1). The culture was incubated overnight (30°C, 160rpm, 16 h). Next day, cells were pelleted (3,200g, 4°C, 10 minutes). The supernatant was discarded and the cells were resuspended in 25 ml periplasmic extraction buffer 1 (20% sucrose, 100 mM Tris-HCl, 1 mM EDTA, pH 8.0). The resuspended cells were incubated on ice for 30 minutes and then centrifuged (10,000g, 4°C, 10 minutes). The supernatant was removed and stored at 4°C- this is "periplasmic fraction-1". The resulting pellet was resuspended in 25 ml periplasmic extraction buffer 2 (5 mM $MgCl_2$) and incubated on ice for 20 minutes. The samples were centrifuged (10,000g, 4°C, 10 minutes) and the supernatant was collected- this is "periplasmic fraction-2". Both the periplasmic fractions were combined, passed through a 0.2-µm filter, and dialyzed overnight at 4°C against 1x phosphate buffered saline (PBS). 1 ml of Ni-NTA resin was added to the dialyzed solution and mixed gently on a rocker (4°C, 1 h). This was transferred into a gravity-flow column and washed with 10 ml PBS buffer. Resin was then washed twice with 8 ml Ni-NTA wash buffer (50 mM Tris-HCl, 300 mM NaCl and 20 mM imidazole, pH 8.0). To elute the bound 7D12, 500 µL Ni-NTA elution buffer (50 mM Tris-HCl, 300 mM NaCl and 200 mM imidazole, pH 8.0) was added and incubated for 15 minutes at room temperature, this was repeated 8 times. Elution fractions were pooled and dialyzed overnight at 4°C against PBS buffer. Dialyzed fractions were then concentrated by Vivaspin 500 columns with 3 kDa molecular weight cut-off (GE Healthcare) and the yields were determined using a calorimetric Pierce BCA protein assay (Thermo Fisher Scientific) measured at 562 nm. After protein purification and concentration, the samples were subsequently resolved by SDS-PAGE. Nu-PAGE LDS loading buffer (Invitrogen) was added to 20 µl of protein samples and heated at 95°C for 15 minutes, then centrifuged (13,000 g, 15 minutes, 4°C) and loaded on a 4-12% Bis-Tris gel (Invitrogen) along with BIO-RAD pre-stained protein ladder (Precision Plus All Blue Protein Standards) as a marker. The gel was then stained with Coomassie Blue (InstantBlue, Expedeon) and the identity of the protein was further confirmed by electrospray ionization mass spectrometry coupled with liquid chromatography, LC-MS. The molecular weights observed on SDS-PAGE gel and mass spectrometry were in good agreement with expected molecular weight of 7D12 and its mutants with site-specifically incorporated photocaged tyrosine (Figure 1B and Figure S5).

On-cell assay for measuring the binding of His-tagged antibody fragments: All cell lines were grown in Dulbecco's modification of Eagle medium (DMEM; Gibco, Invitrogen) containing L-glutamine, 4.5 g/L D-Glucose, 110 mg/L Sodium pyruvate, 10% (v/v) foetal bovine serum (FBS), and 1% (v/v) cocktail of penicillin and streptomycin (PEN/STREP, Sigma-Aldrich), which will be referenced as "complete medium" in the text. A431 and MDA-MB-231 cells were grown in a T-75 flask in complete medium (DMEM, 10% FBS, 1% PEN/STREP) using standard tissue culture procedures until 80-90% confluence. After washing with 1x phosphate buffered saline (PBS) and trypsinising, cells were pelleted (300g, 5 minutes) and resuspended in 10 ml fresh complete medium. The cells were then counted on a hemocytometer and diluted to 200,000 cells/ml. 200 µl of this solution was dispensed into each well (40,000 cells/well) of a 96-well plate and grown overnight. The medium was replaced with 200 µl of complete medium supplemented with 7D12 or its photocaged mutants at the desired concentration. The plate was incubated for 10 minutes (37°C, 5% CO_2). After removing medium, the cells were fixed using formaldehyde; 150 µl of 3.7% formaldehyde solution in sterile Mili-Q water was added to each well and incubated for 20 min at room temperature. The formaldehyde solution was removed and cells were washed three times (200 µl, 5 minutes, gentle rocking) with PBST (1X PBS supplemented with 1% Tween-20). After removing the wash buffer, 100 µl of blocking buffer (10% milk in PBST) was added and cells were incubated at room temperature for 1 h with gentle rocking. The blocking buffer was removed. 50 µl solution containing primary anti-6x-His tag antibody was added to each well and the plate was incubated at room temperature for 1 h. The primary antibody solution contained mouse anti-6x-His tag antibody (ThermoFisher Scientific) at 1:500 dilution and 1% milk in PBST. After incubation with the primary antibody, cells in each well were washed three times with PBST (200 µl, 5 minutes, gentle rocking). Subsequently, 50 µl of HRP-linked secondary antibody solution was applied to each well and incubated at room temperature for 1 h. The HRP-linked secondary antibody solution contained anti-mouse-HRP-linked antibody (Cell Signaling) at 1:1000 dilution and 1% milk in PBST. After incubation with secondary antibody, the cells were washed five times with PBST (200 µl, 5 minutes, gentle rocking). Finally, 200 µl of SuperSignal chemiluminescent Substrate (ThermoFisher Scientific) was added to each well and the plate was imaged using BIORAD GelDoc XR+. The chemiluminescence intensity in each well was further quantified by measuring the signal using a CLARIOstar plate reader (BMG labtech).

Conditions for light-mediated decaging of photoactive antibodies in on-cell assay: In the on-cell assay, wt7D12, 7D12**pcY**32, 7D12**pcY**109 and 7D12**pcY**113 were irradiated with 365 nm light using a UV transilluminator (GelDocMega; BioSystematica).The photon flux and the intensity of 365 nm light from this UV transilluminator, measured using a laser power meter (FieldMate; Coherent), were estimated to be ~33 mW/cm^2 and 14 mW, respectively. 7D12 samples were loaded onto an 18 mm glass coverslip (surface area of coverslip= 2.54cm^2) and placed over the transilluminator, followed by irradiation with 365 nm light for 4 min. After irradiation, samples were transferred to 1.5 ml tube and the concentration of the protein was measured using calorimetric Pierce BCA protein assay (Thermo Fisher Scientific).

Cell viability experiments under irradiation conditions for on-cell assay: 2 ml of A431 cells were seeded (200,000 cell/ml) on 18 mm cover glass placed in a 6-well plate (Corning, product code: 3471). This plate was incubated overnight (37°C, 5% CO_2). The cover glass with the attached cells was removed and placed on a UV transilluminator (GelDocMega; BioSystematica), followed by irradiation with 365 nm light for 4 min. This cover glass was transferred back to complete medium and the cells were allowed to proliferate for 48 h (37°C, 5% CO_2). Subsequently, the cells were detached from the cover glass using trypsin, resuspended in complete media and mixed with resazurin in a 96 well plate. Resazurin (0.1 mg/mL in PBS; Sigma-Aldrich) was added to the cells in a 1:10 dilution for 2 h at 37 °C. Resazurin exhibits a blue colour and low fluorescence in metabolically inactive cells but is converted to a highly fluorescent product (resorufin) upon metabolism by viable cells. Fluorescence was then measured on a Flexstation 3 plate reader (Molecular Devices; laser excitation, 570 nm; emission detection, 600 nm).

Computational methods: The crystal structure for 7D12-EGFR domain III complex (PDB ID: 4KRL)[6] was used as the starting structure for wt7D12-EGFR domain III simulations. This crystal structure was also used to generate the three complexes with the mutants, 7D12**pcY**32, 7D12**pcY**109, and 7D12**pcY**113: **pcY** was modelled in to substitute Y32, Y109, and Y113, respectively. The CHARMM36 force field[7] with CMAP corrections[8] was adopted for proteins and ions, and CGENFF[9] parameters were used for **pcY**. Parameter assignment and optimization for **pcY**, was done using ffTK,[10] following the CGENFF methodology. Quantum mechanical target data for parameter optimization was generated using GAUSSIAN09.[11] Using VMD,[12] each of the four complexes was solvated with water molecules modelled as TIP3P,[13] and Na^+ and Cl^- ions were added to neutralize the system with a net concentration of 150 mM. All the simulations were performed using NAMD 2.12.[14] The systems were minimized for 10,000 steps with all heavy atoms of the protein restrained with a spring constant of k = 5 kcal/mol/Å^2. The restraints on the side chains were then removed and the systems were again minimized for 10,000 steps, followed by equilibration for 0.2 ns using the NVT ensemble, with stepwise heating up to 310 K, to allow the side chains to relax. Next, all the restraints were removed and the systems were again minimized for 10000 steps, then equilibrated for 0.2 ns in the NVT ensemble with stepwise heating to 310K. Finally, the system was equilibrated in the NPT ensemble for 0.5 ns, followed by the data collection runs for 300 ns using the NPT ensemble and periodic boundary conditions. Temperature was maintained at 310 K using Langevin dynamics[15] with a damping coefficient of 1 ps^{-1}. Pressure was kept at 1 atm using the Nosé-Hoover Langevin piston method[15-16] with a piston period of 100 fs and a piston decay of 50 fs. Short-range interactions were cut off at 12 Å with a switching applied at 10 Å. Long-range electrostatic forces were calculated using the particle mesh Ewald (PME)[17] method at a grid density of >1 Å^{-3}. Bonded, non-bonded, and PME calculations were performed at 2-, 2-, and 4-fs intervals, respectively.

Labelling of antibody fragments with BODIPY-TMR-X: To a 37.5 µl solution of wt7D12 (100 µM) or 7D12**pcY**32 (100 µM), 51 µl of water, 1.5 µl of 10 mM BODIPY-TMR-X SE in DMF (ThermoFisher Scientific) and 10 µl of 1 M $NaHCO_3$ (pH 8) were added. The reaction mixture was incubated at 25°C for 1 h with shaking (600rpm). To remove excess fluorophore, the labelled wt7D12 and 7D12**pcY**32 samples were applied to Zeba desalting columns (MWCO 7000 Da, ThermoFisher Scientific). To ensure that all the unbound fluorophore is removed, desalting was performed three times for each sample. The concentration of wt7D12-TMR-X and 7D12**pcY**32-TMR-X were determined using a calorimetric Pierce BCA protein assay (ThermoFisher Scientific) measured at 562 nm.

Microscopy set-up: For all live cell imaging experiments, a physiological imaging medium was used to provide better clarity and reduced background. This imaging medium contained 120 mM NaCl, 5 mM KCl, 2 mM $CaCl_2.2H_2O$, 1 mM $MgCl_2.6H_2O$, 1 mM NaH_2PO_4, 1 mM $NaHCO_3$, 25 mM HEPES and adjusted to pH 7.2. Once the pH was adjusted, medium was supplemented with 11 mM Glucose, 2.5 mM myo-Inositol, 2 mM Glutamine and BME amino acids (50X solution from Sigma-Aldrich). This medium will be referenced as “imaging medium” in the text. 2 ml of A431 cells (400,000 cells/well) suspended in complete medium were seeded in each well of an ultra-low attachment 6-well plate (Corning, 3471) containing 18 mm cover glass. This plate was incubated overnight (37°C, 5% CO_2). Once 80-90% confluence was observed, cover glass was washed three times with imaging medium and mounted into a microscope chamber (Luidin chamber, Life imaging services). A peristaltic pump was connected to the microscope chamber and allowed for a constant flow of imaging media over live cells at a rate of 1 ml/min. Time-lapse was set up to take brightfield and TexasRed (500ms exposure) images every 30 seconds over a 42 min time period. Cells were imaged on a Zeiss Axiovert 200M microscope at 37ºC using Zeiss AxioVision software. Brightfield and fluorescence images were captured using a 40x (1.3 NA) Plan-Neofluar, oil-immersion objective lens and a Zeiss AxioCam MRm CCD camera. BODIPY-TMR-X fluorescence was excited at 572 ± 14 nm and emission collected at 629 ± 28 nm. 500nM dilutions of wt7D12 and 7D12**pcY**32 were made up in imaging medium and passed over A431 cells at different time points. First, 2 ml of 7D12**pcY**32 was passed over with no 365 nm light, followed by 10 ml wash of imaging medium. Then another 2 ml of 7D12**pcY**32 was passed over while being irradiated with 365 nm for 1 minute. After another wash of 10 ml imagine medium, 2 ml of wt7D12 was passed, followed by the final wash. Microscopy images were processed by ImageJ (Fiji).

Conditions for light-mediated decaging of photoactive antibodies in microscopy experiments: Light-mediated activation of 7D12**pcY**32 on the microscope was achieved by opening DAPI channel for 1 minute. The photon flux and the laser power of 365 nm light from DAPI channel, measured using laser power meter (FieldMate; Coherent), were estimated to be ~0.25 mW/cm^2 (40x 1.3 NA Plan-Neofluar objective area of 3.06mm^2) and 8 mW, respectively.

Cell viability experiments under irradiation conditions for microscopy experiments: 2 ml of A431 cells were seeded (400,000 cell/well) on 18 mm cover glass placed in a 6-well plate (Corning, product code: 3471). This plate was incubated overnight (37°C, 5% CO_2). The cover glass with the attached cells was removed and placed under the microscope, followed by irradiation with 365 nm light for 1 min. This cover glass was transferred back to complete medium and the cells were allowed to proliferate for 48 h (37°C, 5% CO_2). Subsequently, the cells were detached from the cover glass using trypsin, resuspended in complete media and mixed with resazurin in a 96 well plate. Resazurin (0.1 mg/mL in PBS; Sigma-Aldrich) was added to the cells in a 1:10 dilution for 2 h at 37 °C. Resazurin exhibits a blue colour and low fluorescence in metabolically inactive cells but is converted to a highly fluorescent product (resorufin) upon metabolism by viable cells. Fluorescence was then measured on a Flexstation 3 plate reader (Molecular Devices; laser excitation, 570 nm; emission detection, 600 nm).

Figure S1. Sequence of gene block inserted into pSANG10 plasmid for generation of pSANG10_7D12 plasmid. Red – Gibson overhang, and Underlined – Restriction sites used for cloning. Protein sequence is shown below the DNA sequence, Green – leader peptide sequence, Purple – wt7D12 sequence, Blue – His6- tag, and black Y* - sites for insertion of **pcY**

```
ttaactttaagaaggagatatacatatgaaatcgctgattactccgattgctgccggatta
                                  M  K  S  L  I  T  P  I  A  A  G  L
ctgttggcgttttcccaatactcgttggcgcaggtaaaattagaagaatctgggggtggg
 L  L  A  F  S  Q  Y  S  L  A  Q  V  K  L  E  E  S  G  G  G
tccgttcagacgggtggaagtctgcgcttgacatgtgccgcgtctgggcgcacatcacgc
 S  V  Q  T  G  G  S  L  R  L  T  C  A  A  S  G  R  T  S  R
tcatatggaatgggctggttccgtcaagctcctggcaaggagcgtgagttcgtatccggt
 S  Y* G  M  G  W  F  R  Q  A  P  G  K  E  R  E  F  V  S  G
atctcatggcgcggtgactcaaccggatatgctgactcagtaaagggggcgtttcaccatc
 I  S  W  R  G  D  S  T  G  Y  A  D  S  V  K  G  R  F  T  I
tctcgtgataatgcgaagaatactgtcgacttacaaatgaactcacttaaaccagaggat
 S  R  D  N  A  K  N  T  V  D  L  Q  M  N  S  L  K  P  E  D
accgctatttactactgcgctgctgcggcagggtctgcatggtacggtacactgtacgag
 T  A  I  Y  Y  C  A  A  A  A  G  S  A  W  Y  G  T  L  Y* E
tacgactactgggggcagggcacccaggtcacggtttcttctcaccaccatcaccaccac
 Y D  Y*  W  G  Q  G  T  Q  V  T  V  S  S  H  H  H  H  H  H
tgataaaagctttaataagtcgagcac
```

Figure S2. Expression of wt7D12 using pSANG10_7D12 plasmid. A) Coomassie stained gel image demonstrate that the expression of 7D12 is dependent on addition of IPTG. B) ESI-MS of the protein 7D12 confirms its identity.

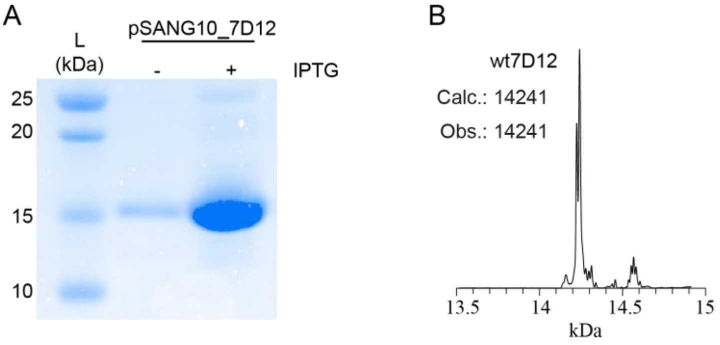

Figure S3. Screening of pSUP_*Mj*CNFRS/6x*Mj*tRNA$_{CUA}$, pEvolv_2x*Mj*CNFRS/*Mj*tRNA$_{CUA}$ and pULTRA_*Mj*CNFRS/*Mj*tRNA$_{CUA}$ plasmids. Comparison of band intensities in the western blot demonstrates unnatural amino acid dependent expression of 7D12 and that the pULTRA suppressor plasmid is most efficient for incorporation of 4-Azido-L-phenylalanine (AzF) using *Mj*RS/*Mj*tRNA pair in 7D12.

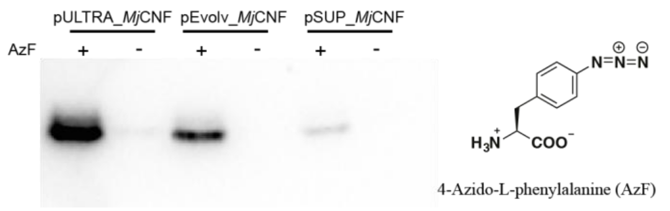

Figure S4. Genetic site-specific incorporation of N6-(tert-butoxycarbonyl)-L-lysine (BocK) and N6-[(2-propynyloxy)carbonyl]-L-lysine (CAK) in 7D12. Comparison of band intensities in the Coomassie stained gel demonstrates that pCDF suppressor plasmid is more efficient than the pULTRA suppressor plasmid at incorporating unnatural amino acids using PylRS/PyltRNA pair.

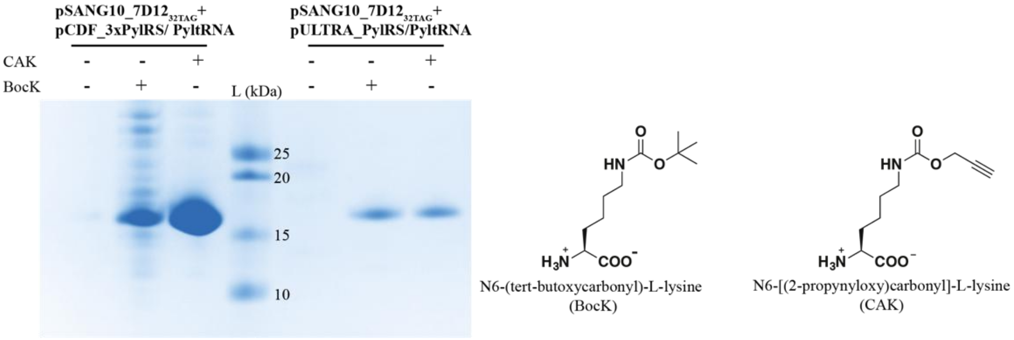

Figure S5. ESI-MS of the wt7D12, 7D12**pcY**32, 7D12**pcY**109, and 7D12**pcY**113 confirms their identity. The observed and the calculated molecular weights are in good agreement with each other.

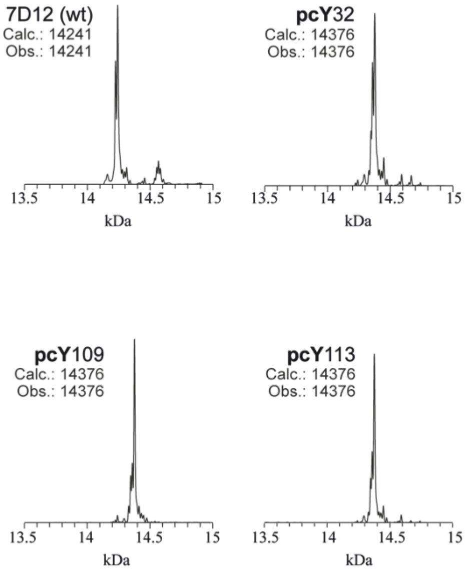

Figure S6. On-cell assay reports specific binding between 7D12 antibody fragment and EGFR on cell surface. Low chemiluminescence signal observed when wt7D12 was incubated with MDA-MB-231 cells, a cell line with low levels of EGFR, compared to the signal from A431 cancer cell line, that has higher levels of EGFR.

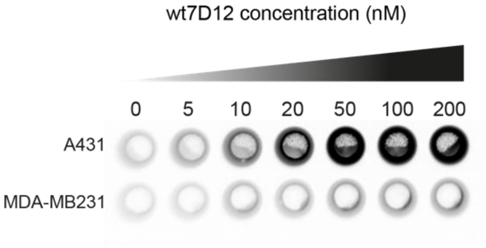

Figure S7. Non-EGFR binding His6-tagged antibody fragments do not bind to the surface of A431 cells. Near background level chemiluminescence signal observed when an unrelated His6-tagged antibody fragment, RR6-VHH, is incubated with A431 cancer cells.

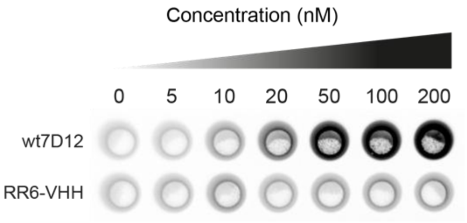

Figure S8. On-cell binding experiments to assess the interaction of EGFR with mutants of 7D12 containing site-specifically incorporated **pcY** were performed in triplicates. On-cell assays performed on the surface of A431 cells demonstrates that the presence of **pcY** at positions 32 and 113 in 7D12 inhibits its binding to EGFR. However, 7D12**pcY**109 mutant shows binding affinity similar to wt7D12. The binding to 7D12**pcY**32 and 7D12**pcY**113 mutants is restored upon irradiation with 365 nm light. These experiments were performed in triplicates, REP1, REP2 and REP3, to ensure reproducibility of data.

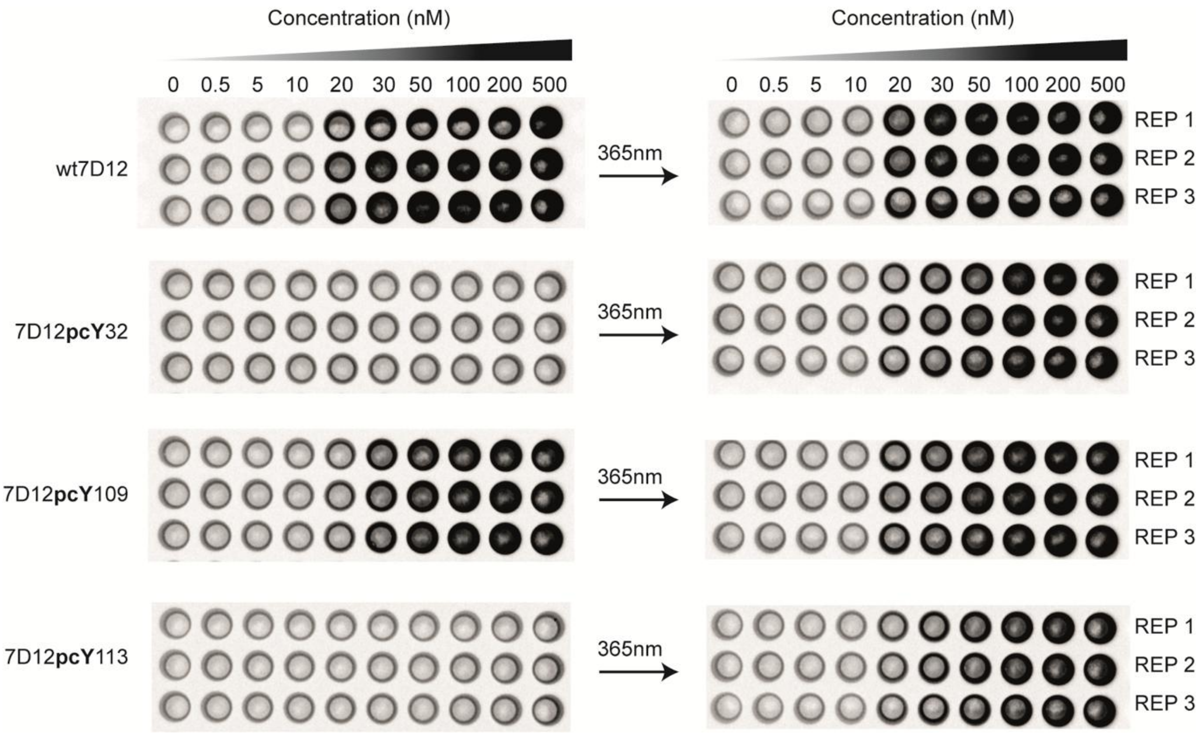

Figure S9. Quantitative assessment of light-mediated binding of photoactive 7D12 to EGFR on the surface of A431 cancer cells. A) Chemiluminescence intensities obtained from on-cell binding experiments for wt7D12, 7D12**pcY**32, 7D12**pcY**109 and 7D12**pcY**113, before and after irradiation with 365 nm light, were quantified using CLARIOstar plate reader and concentration of 7D12, where X-axis is in log scale. The lines show the trace obtained after fitting the data to a sigmoidal nonlinear equation using GraphPad. Some error bars are too small to be clearly visible. B) The K_D and I_{MAX} values obtained for 7D12 and its mutants containing **pcY** after fitting the data. K_D is the concentration of the 7D12, where chemiluminescence intensity is half of the maximum chemiluminescence intensity. I_{MAX} is the maximum chemiluminescence intensity at saturation. The value of I_{MAX} for decaged 7D12**pcY**32 and 7D12**pcY**113 is 86% and 76%, respectively, of the I_{MAX} for wt7D12; indicating recovery of binding to these levels.

A)

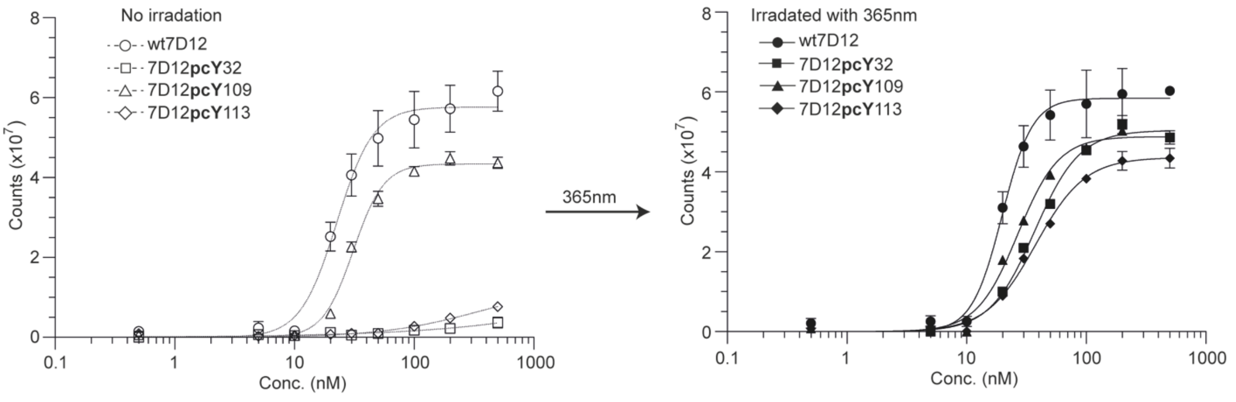

B)

	WT -	WT +	Y32 -	Y32 +	Y109 -	Y109 +	Y113 -	Y113 +
K_D (nM)	23 (±2.6)	20 (±1.8)	N/A	37 (± 2.6)	31 (±1.5)	27 (±1.6)	N/A	38 (±2.6)
I_{max}	5.8 (±0.33) x10^7	5.8 (±0.28) x10^7	N/A	5.0 (±0.17) x10^7	4.3 (±0.12) x10^7	4.9 (±0.14) x10^7	N/A	4.4 (±0.14) x10^7
R^2	0.97	0.98	N/A	0.99	0.99	0.99	N/A	0.99

Figure S10. Measurement of cell viability of A431 cells after irradiation with 365 nm light on UV transilluminator. A431 cells were irradiated with 365 nm light for 4 minutes on a UV transilluminator; similar irradiation conditions were used for decaging 7D12 in on-cell experiments shown in Figure 2. Cells were allowed to proliferate for 48 hours after irradiation with 365 nm light, and viability of these cells was measured using Resazurin cell viability assay. A decrease of ~12% in A431 cell viability is observed after irradiation with 365 nm light. This experiment was performed in duplicates.

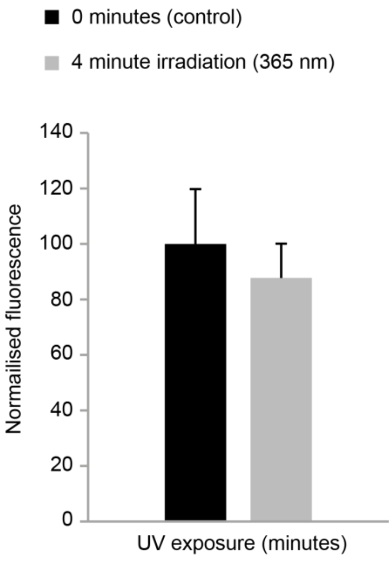

Figure S11. The number of 7D12-EGFR domain III contacts during 300ns of simulations, measured as the number of 7D12 heavy atoms within 3.5 Å of EGFR heavy atoms, show that 7D12**pcY**32 and 7D12**pc**Y113 form lesser contacts with EGFR for long periods, compared to the complexes with wt7D12 and 7D12**pcY**109. These observations suggest that the presence of **pcY** at positions 32 and 113 destabilizes the 7D12-EGFR domain III complex.

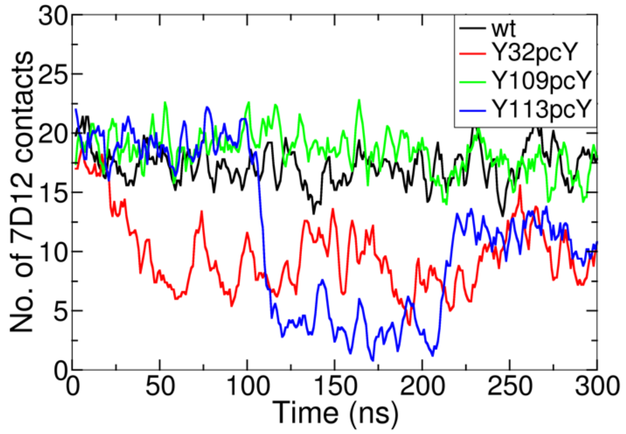

Figure S12. The binding of 7D12 to EGFR is affected by presence of BODIPY-TMR-X label. Comparison of binding of labelled and unlabelled wt7D12 to EGFR assessed using on-cell assay on the surface of A431 cells shows a reduction in binding by ~1.5-fold due to the presence of BODIPY-TMR-X label. The chemiluminescence counts for wt7D12 and labelled-7D12 at 200 nM concentration are 6838468 and 4436744, respectively.

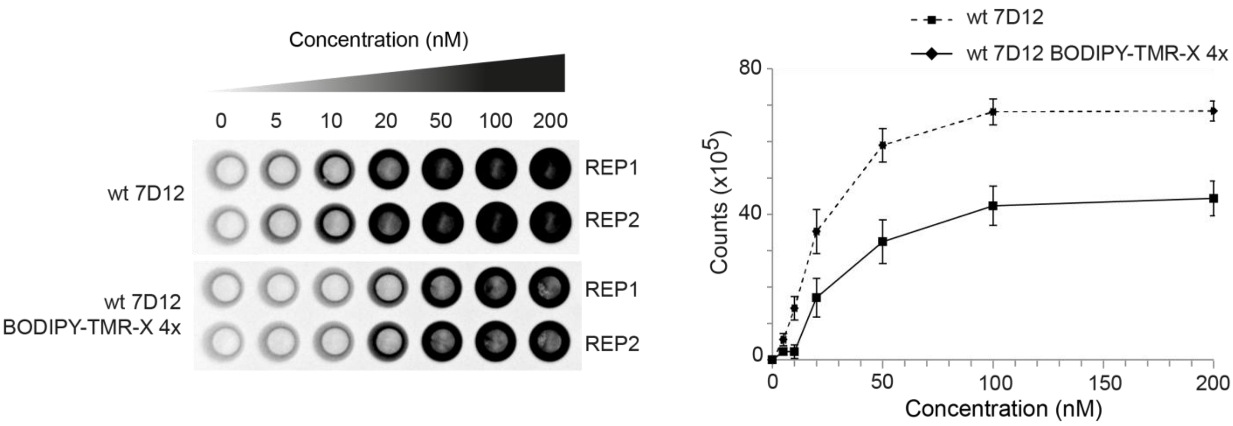

Figure S13. Measurement of cell viability of A431 cells after irradiation with 365 nm light on the microscope. A431 cells were irradiated for 1 minute by opening the DAPI channel on microscope; similar irradiation conditions were used in microscopy experiments shown in Figure 4. Cells were allowed to proliferate for 48 hours after irradiation with 365 nm light, and viability of these cells was measured using Resazurin cell viability assay. A decrease of ~13% in A431 cell viability is observed after irradiation with 365 nm light.

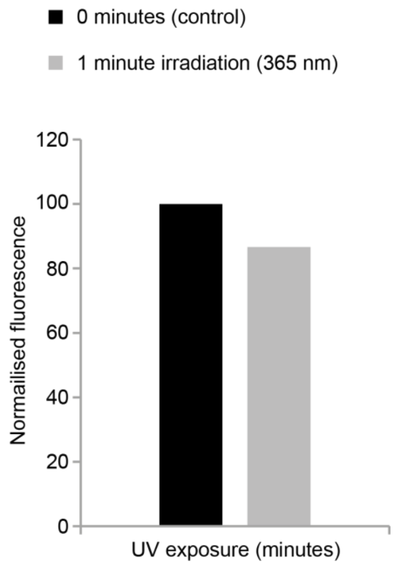

References

[1] a) A. Deiters, D. Groff, Y. Ryu, J. Xie, P. G. Schultz, *Angew. Chem. Int. Ed. Engl.* **2006**, *45*, 2728-2731; b) D. P. Nguyen, H. Lusic, H. Neumann, P. B. Kapadnis, A. Deiters, J. W. Chin, *J. Am. Chem. Soc.* **2009**, *131*, 8720-8721.
[2] a) G. Biffi, D. Tannahill, J. McCafferty, S. Balasubramanian, *Nat. Chem.* **2013**, *5*, 182-186; b) C. D. Martin, G. Rojas, J. N. Mitchell, K. J. Vincent, J. Wu, J. McCafferty, D. J. Schofield, *BMC Biotechnol.* **2006**, *6*, 46.
[3] Y. Ryu, P. G. Schultz, *Nat. Methods* **2006**, *3*, 263-265.
[4] T. S. Young, I. Ahmad, J. A. Yin, P. G. Schultz, *J. Mol. Biol.* **2010**, *395*, 361-374.
[5] A. Chatterjee, S. B. Sun, J. L. Furman, H. Xiao, P. G. Schultz, *Biochemistry* **2013**, *52*, 1828-1837.
[6] K. R. Schmitz, A. Bagchi, R. C. Roovers, P. M. van Bergen en Henegouwen, K. M. Ferguson, *Structure* **2013**, *21*, 1214-1224.
[7] A. D. MacKerell, D. Bashford, M. Bellott, R. L. Dunbrack, J. D. Evanseck, M. J. Field, S. Fischer, J. Gao, H. Guo, S. Ha, D. Joseph-McCarthy, L. Kuchnir, K. Kuczera, F. T. Lau, C. Mattos, S. Michnick, T. Ngo, D. T. Nguyen, B. Prodhom, W. E. Reiher, B. Roux, M. Schlenkrich, J. C. Smith, R. Stote, J. Straub, M. Watanabe, J. Wiorkiewicz-Kuczera, D. Yin, M. Karplus, *J. Phys. Chem. B* **1998**, *102*, 3586-3616.
[8] A. D. Mackerell, Jr., M. Feig, C. L. Brooks, 3rd, *J. Comput. Chem.* **2004**, *25*, 1400-1415.
[9] K. Vanommeslaeghe, E. Hatcher, C. Acharya, S. Kundu, S. Zhong, J. Shim, E. Darian, O. Guvench, P. Lopes, I. Vorobyov, A. D. Mackerell, Jr., *J. Comput. Chem.* **2010**, *31*, 671-690.
[10] C. G. Mayne, J. Saam, K. Schulten, E. Tajkhorshid, J. C. Gumbart, *J. Comput. Chem.* **2013**, *34*, 2757-2770.
[11] M. J. Frisch, G. W. Trucks, H. B. Schlegel, G. E. Scuseria, M. A. Robb, J. R. Cheeseman, G. Scalmani, V. Barone, G. A. Petersson, H. Nakatsuji, X. Li, M. Caricato, A. V. Marenich, J. Bloino, B. G. Janesko, R. Gomperts, B. Mennucci, H. P. Hratchian, J. V. Ortiz, A. F. Izmaylov, J. L. Sonnenberg, Williams, F. Ding, F. Lipparini, F. Egidi, J. Goings, B. Peng, A. Petrone, T. Henderson, D. Ranasinghe, V. G. Zakrzewski, J. Gao, N. Rega, G. Zheng, W. Liang, M. Hada, M. Ehara, K. Toyota, R. Fukuda, J. Hasegawa, M. Ishida, T. Nakajima, Y. Honda, O. Kitao, H. Nakai, T. Vreven, K. Throssell, J. A. Montgomery Jr., J. E. Peralta, F. Ogliaro, M. J. Bearpark, J. J. Heyd, E. N. Brothers, K. N. Kudin, V. N. Staroverov, T. A. Keith, R. Kobayashi, J. Normand, K. Raghavachari, A. P. Rendell, J. C. Burant, S. S. Iyengar, J. Tomasi, M. Cossi, J. M. Millam, M. Klene, C. Adamo, R. Cammi, J. W. Ochterski, R. L. Martin, K. Morokuma, O. Farkas, J. B. Foresman, D. J. Fox, Wallingford, CT, **2016**.
[12] W. Humphrey, A. Dalke, K. Schulten, *J. Mol. Graph.* **1996**, *14*, 33-38, 27-38.
[13] W. L. Jorgensen, J. Chandrasekhar, J. D. Madura, R. W. Impey, M. L. Klein, *J. Chem. Phys.* **1983**, *79*, 926-935.
[14] J. C. Phillips, R. Braun, W. Wang, J. Gumbart, E. Tajkhorshid, E. Villa, C. Chipot, R. D. Skeel, L. Kale, K. Schulten, *J. Comput. Chem.* **2005**, *26*, 1781-1802.
[15] G. J. Martyna, D. J. Tobias, M. L. Klein, *J. Chem. Phys.* **1994**, *101*, 4177-4189.
[16] S. E. Feller, Y. H. Zhang, R. W. Pastor, B. R. Brooks, *J. Chem. Phys.* **1995**, *103*, 4613-4621.
[17] T. Darden, D. York, L. Pedersen, *J. Chem. Phys.* **1993**, *98*, 10089-10092.

Author Contributions

Thomas Bridge performed all primary lab experiments and data analysis, and assisted in writing the paper. Saher A. Shaikh performed computational studies, wrote the sections on computational studies, and assisted in lab experiments and in writing the paper. Paul Thomas provided training and support for microscopy experiments. Joaquin Botta provided initial training in on-cell experiments. Peter J. McCormick provided A431 cells and initial assistance in on-cell experiments. Amit Sachdeva conceived the idea, designed experiments, provided resources, performed lab experiments, and wrote the paper.
